# Flagellin outer domain dimerization modulates motility in pathogenic and soil bacteria from viscous environments

**DOI:** 10.1038/s41467-022-29069-y

**Published:** 2022-03-17

**Authors:** Mark A. B. Kreutzberger, Richard C. Sobe, Amber B. Sauder, Sharanya Chatterjee, Alejandro Peña, Fengbin Wang, Jorge A. Giron, Volker Kiessling, Tiago R. D. Costa, Vincent P. Conticello, Gad Frankel, Melissa M. Kendall, Birgit E. Scharf, Edward H. Egelman

**Affiliations:** 1 Department of Biochemistry and Molecular Genetics, University of Virginia School of Medicine, Charlottesville, VA 22903 USA; 2 Department of Biological Sciences, Virginia Tech, Blacksburg, VA 24061 USA; 3 Department of Microbiology, Immunology, and Cancer Biology, University of Virginia School of Medicine, Charlottesville, VA 22903 USA; 4 MRC Centre for Molecular Bacteriology and Infection, Department of Life Sciences, Imperial College, London, SW7 2AZ UK; 5 Department of Pediatrics, University of Virginia School of Medicine, Charlottesville, VA 22903 USA; 6 Department of Molecular Physiology and Biological Physics, University of Virginia School of Medicine, Charlottesville, VA 22903 USA; 7 Department of Chemistry, Emory University, Atlanta, GA 30322 USA

**Keywords:** Molecular conformation, Cryoelectron microscopy

## Abstract

Flagellar filaments function as the propellers of the bacterial flagellum and their supercoiling is key to motility. The outer domains on the surface of the filament are non-critical for motility in many bacteria and their structures and functions are not conserved. Here, we show the atomic cryo-electron microscopy structures for flagellar filaments from enterohemorrhagic *Escherichia coli* O157:H7, enteropathogenic *E. coli* O127:H6, *Achromobacter*, and *Sinorhizobium meliloti*, where the outer domains dimerize or tetramerize to form either a sheath or a screw-like surface. These dimers are formed by 180° rotations of half of the outer domains. The outer domain sheath (ODS) plays a role in bacterial motility by stabilizing an intermediate waveform and prolonging the tumbling of *E. coli* cells. Bacteria with these ODS and screw-like flagellar filaments are commonly found in soil and human intestinal environments of relatively high viscosity suggesting a role for the dimerization in these environments.

## Abstract (web summary)

It has been suggested that the outer domains of bacterial flagellins are not needed for motility. Here, the authors show that flagellar filament outer domains from some bacteria have unique structures which can alter the motility of the bacteria.

## Introduction

Bacteria use their flagella to swim towards or away from various environmental signals^1^. The flagellum is divided into several parts: the motor (basal body), the rod, the hook, and the filament^2,3^. The rotating flagellar filament primarily functions as a propeller, acting as an Archimedean screw, and supercoiling of the filament is essential as rotations of a straight filament do not generate thrust.

Bacterial species from the *Escherichia* and *Salmonella* genera have peritrichous flagella^4^. During running mode, the flagellar motors at the base of the flagellum are rotating counterclockwise, which orients the filaments in a bundle toward one end of the cell^5,6^. When the direction of rotation of one or several flagella changes clockwise, the affected flagellar filaments break out of the bundle, and the filament switches from the normal left-handed waveform to right-handed semi-coiled and curly forms^6,7^. This causes the cell to tumble, allowing the trajectory of the cell to change^5–9^. During chemotaxis, the alternation between running and tumbling modes depends on the presence of both positive and negative stimuli^10^ and result in a “biased random-walk” swimming pattern^11^.

The mechanism of flagellar filament supercoiling has been attributed to the existence of the 11 protofilaments in a mixture of two subunit conformations that differ very slightly in length^12,13^. The short protofilaments are located on the inside of the supercoil curve, while the long protofilaments are on the outside. It is this path length difference between the short and long protofilaments that causes the supercoiling. Most previous structural studies analyzed straight mutant flagellar filaments so that helical symmetry could be imposed for the purposes of averaging^13–16^. Recently, high-resolution cryo-EM structures of supercoiled flagellar filaments were achieved with helical reconstruction, assuming all the protofilaments are in a single state^17,18^. These studies provided valuable information related to flagellar filament structure, but very few insights into the mechanism of polymorphic switching. However, they showed that a high-resolution cryo-EM structure can be generated without using straight mutant flagellar filaments.

The polymorphic switching of the flagellar filament has been attributed to the N- and C-terminal core domains D0 and D1 of flagellin^12,13,19,20^. The central flagellin region contains the “hyper-variable” outer domains, which have been shown to have non-critical roles in motility for some bacterial species^13,21–23^. Large deletions of up to 100 amino acid residues in the outer domains D2 and D3 in *Salmonella typhimurium* flagellin FliC had no effect on bacterial motility^21,22^. In addition, many bacterial species have flagellar filaments assembled from flagellins with just the core domains D0 and D1 and no outer domains^13,17,24^. Rather than affecting motility, many outer domains are thought to provide their flagellar filaments with non-conserved species-specific functions such as adhesion^25–27^, modulation of host immune responses^28–31^, or protease activity^32^.

The flagellar filaments of certain soil bacteria such as *Sinorhizobium meliloti* have been called “complex” due to the pairing of outer domain subunits to produce a criss-cross pattern on the surface of the filament^33–35^. This pairing was regarded as a perturbation of the underlying symmetry^35^, modeled in part on the work done with the Dahlemense strain of tobacco mosaic virus (TMV)^36^. This model proposed that pairing of subunits across the helical groove, involving displacements of ~3 Å, served to break the helical symmetry. In TMV, subunits are rigidly locked into a helical lattice and major rearrangements of subunits would not be possible. We discovered the existence of non-canonical flagellar filaments in *Achromobacter* sp. MFA1 R4^37,38^, a soil bacterium that was identified as a cell culture contaminant. These flagellar filaments have a sheath surrounding the core of the filament similar to those previously described for some H-serotypes of *E. coli*^39,40^.

Here, we show the high-resolution cryo-EM structures of the *S. meliloti*, enterohemorrhagic *E. coli* O157:H7 (EHEC O157:H7)*, Achromobacter,* and enteropathogenic *E. coli* O127:H6 (EPEC O127:H6) flagellar filaments. The three former structures would have been classified as “helically perturbed” while the latter as “non-helically perturbed”^41,42^. In the non-helical perturbation model, subunit pairing introduced a seam causing a discontinuity in the helical lattice^41^, much as such a seam or discontinuity exists in microtubules^43^. In the present study, we show that the outer domains in these flagellar filaments have considerable freedom. In physics and mathematics, perturbations are regarded as small deviations, such as the influence of the gravitational field of the sun on the orbit of the moon around the earth^44^. Instead of the previously predicted small perturbations, half of the outer domain population rotated by 180° to form symmetrical dimers or tetramers with other outer domains. These outer domain dimers form either a screw-like surface surrounding the filament core domains (D0 and D1) as in *S. meliloti*, or an outer domain sheath (ODS) around the core as in EHEC O157:H7, *Achromobacter* sp, and EPEC O127:H6.

The ODS surrounding the EHEC H7 and *Achromobacter* flagellar filaments produces an intermediate waveform that is not adopted by the “canonical” *S. typhimurium* and *E. coli* K-12 H48 flagellar filaments. In addition, we provide evidence that the ODS of the EHEC H7 flagellar filament prolongs the average time *E. coli* cells spent in the tumbling mode and suggest that this behavior is due to an additional step in flagellar polymorphism during tumbling created by the intermediate waveform. We hypothesize that extended tumbling mediated by ODS flagellar filaments offers an advantage to both intestinal pathogens and soil bacteria by allowing for better reorientation of cells in their respective environments.

## Results

### Cryo-electron microscopy structures of screw-like and ODS flagellar filaments

**Fig. 1:**
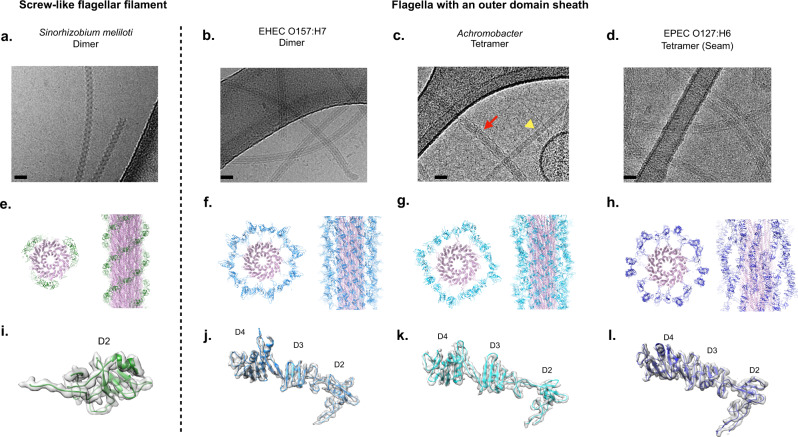
Cryo-EM structure of outer-domain sheath and screw-like flagellar filaments. **a**–**d** Cryo-electron micrographs of *S. meliloti* (**a**), EHEC H7 (**b**), *Achromobacter* (**c**), and EPEC H6 (**d**) flagellar filaments. The scale bar in each micrograph is ~20 nm. In **c**, the red arrow points to an *Achromobacter* flagellar filament. The yellow triangle indicates an *Agrobacterium tumefaciens* flagellar filament. Scale bar is 200 Å. Atomic models of the *S. meliloti* (**e**), EHEC H7 (**f**), *Achromobacter* (**g**), and EPEC H6 flagellar filaments (**h**). The conserved D0/D1 core domains are shown in pink. Outer domains are colored green for the screw-like flagellar filament (**e**) and various shades of blue for those with an outer-domain sheath **f**–**h**. Models and density maps for the single outer domain (D2) of *S. meliloti* (**i**), and for the three outer domains (D2, D3, D4) of EHEC H7 (**j**), *Achromobacter* (**k**), and EPEC H6 (**l**).

Cryo-electron microscopy (cryo-EM) was used to determine the structures of flagellar filaments from *Sinorhizobium meliloti*, enterohemorrhagic *E. coli* O157:H7 (EHEC O157:H7), *Achromobacter*, and enteropathogenic *E. coli* O127:H6 (EPEC O127:H6). Differences in filament surface structures were observable in the cryo-electron micrographs by the naked eye (Fig. 1a–d). The power spectrum of each flagellar filament (Supplementary Fig. 1b–e) was very different from that of any published canonical flagellar filament (Supplementary Fig. 1a)^13^, indicative of the uniqueness of each structure.

The *S. meliloti*, EHEC O157:H7, and *Achromobacter* flagellar filament structures were solved using helical reconstruction to 3.7, 3.6, and 3.7 Å resolution, respectively (Supplementary Table 1). Since a seam breaks the helical symmetry in the EPEC O127:H6 flagellar filament, reconstruction was performed asymmetrically to 4.0 Å. Clear side-chain densities and well-resolved β-sheets were observed for the flagellin subunits of each filament (Supplementary Fig. 2). For the *S. meliloti* flagellar filament, a prominent 3-start helix is created by the outer domains surrounding the flagellar core (Fig. 1e), which gives the *S. meliloti* filament surface the appearance of a 3-start screw. A simple screw can be described by a 1-start helix, where there is a single continuous helix forming the threads on the surface of the screw. For a 3-start screw, there are three separate strands that form the surface threads. The outer domains of the EHEC O157:H7, *Achromobacter*, and EPEC O127:H6 flagellar filaments form a sheath-like structure surrounding the core domains D0 and D1 (Fig. 1f–h), which we named an ODS. The *S. meliloti* filament possesses a single outer domain, D2 (Fig. 1i), while the ODS filaments have three outer domains, D2, D3 and D4 (Fig. 1j–l). The folds of the sheath-forming outer domains D2 and D3 have no homology or similarity to those of the D2 and D3 domains in *Salmonella* filaments^15^.

A shared feature in all four structures is the formation of symmetrical homodimers between the outer domains of flagellin subunits, with one outer domain rotated 180° relative to the other (Supplementary Fig. 3). These symmetrical dimers are the result of outer domains being able to adopt either an “up” or “down” conformation. The outer surface of all these filaments is therefore bipolar. This shared feature is surprising as while the structures and sequences of the EHEC H7, *Achromobacter*, and EPEC H6 flagellin outer domains are all similar, the *S. meliloti* outer domains shared no sequence homology (Supplementary Fig. 4) or structural homology (Fig. 1i–l) with the others.

### Dimeric outer domain interactions of the *S. meliloti* screw-like filament

**Fig. 2:**
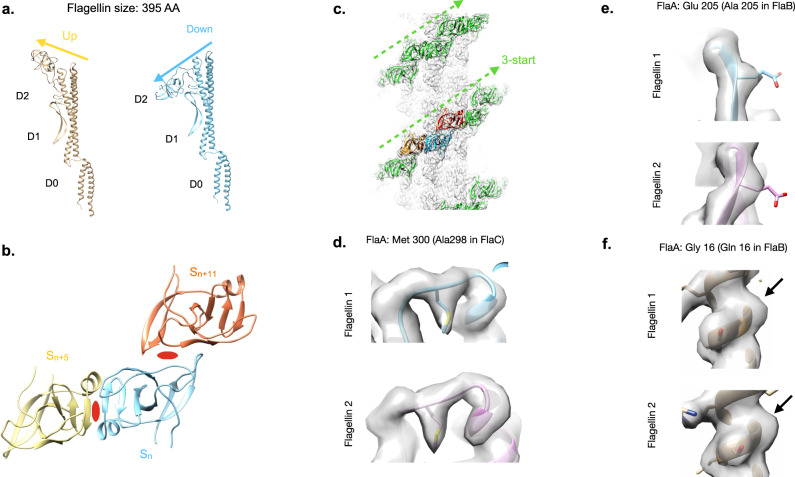
Structural details of *S. meliloti* screw-like flagellar filament. **a** The two flagellin conformations of the *S. meliloti* flagellar filament asymmetric unit. The core domains D0 and D1 are unchanged in either conformation. The outer domains however change and have either an up or down conformation. **b** Each *S. meliloti* outer domain from subunit S_n_ interacts with the outer domains of flagellins that are 5 (S_n + 5_) and 11 (S_n + 11_) subunits away. Two-fold axes are indicated by the red symbols. **c** The S_n_:S_n + 5_ and S_n_: S_n + 11_ dimers generate a right-handed 3-start helix (green) surrounding the filament core (light gray). **d**. Model and map showing methionine 300 for the FlaA model fitting the map density well for both flagellin conformations. **e** FlaA model and map density of position 205 for both flagellins in the ASU. **f** FlaA model and map density of position 16 for both flagellins in the ASU.

The asymmetric unit (ASU) of the *S. meliloti* flagellar filament consists of two flagellin conformations, which are identical in the core domains, but the outer domains are rotated 180° from each other resulting in an “up” and a “down” conformation (Fig. 2a). The D2 domains in the *S. meliloti* filament form dimers between an arbitrary “up” conformation subunit (S_n_) and two “down” conformation subunits (S_n + 5_) and (S_n + 11_) as depicted in Fig. 2b. Since the outer surface is bipolar, a “down” D2 will also interact with two “up” D2s. These interactions form a 3-start helix surrounding the flagellar core, which resembles the grooves in a helical screw (Fig. 2c). They can be described as a reduction in helical symmetry along the 6-start helix, resulting in a 3-start helix (Supplementary Fig. 5a). Due to this subunit pairing, the axial rise and helical twist of the outer domains are twice those of the flagellar core (Table 1).

**Table 1:** Helical symmetry parameters for *S. meliloti*, EHEC H7, *Achromobacter* sp. MFA1 R4, and EPEC H6.

Organism	Core rise (Å)^a^	Core twist (°)^a^	Outer domain rise (Å)	Outer domain twist (°)	Outer domain symmetry^b^
*Sinorhizobium meliloti*	4.75	65.46	9.5	130.92	Dimer
EHEC O157:H7	4.8	65.4	9.6	130.8	Dimer
*Achromobacter*	4.725	65.4	18.9	−98.4	Tetramer
EPEC O127:H6^c^	4.813	65.534	N/A	N/A	Tetramer w/seam

^a^The core domain rise and twist are the helical parameters of core domains D0 and D1 for each filament.

^b^The outer domain symmetry indicates the symmetry used to reconstruct the full flagellar filament.

^c^Because of the seam in EPEC H6, there is no overall helical symmetry that can reconstruct the entire filament. However, the core (comprised of domains D0/D1) displays a canonical flagellar filament symmetry.

It has been hypothesized that flagellin dimers in *S. meliloti* are comprised of one each of FlaA and FlaX where FlaX can be any of the other three flagellins^45^. A multiple sequence alignment of all four *S. meliloti* flagellins reveals key similarities and differences among them (Supplementary Fig. 6). FlaD is composed of 321 amino acid residues, and therefore is too short to fit the structure. In addition, it is transcribed at very low levels and expected to have a minor structural function^45^. FlaA, B, and C have the correct size, but a few key residues in FlaC clearly do not fit the density map (Fig. 2d). FlaA and FlaB are very similar in sequence. In positions with residue variations between FlaA and FlaB, there is support for FlaA at position 205 (Fig. 2e) and FlaB at position 16 (Fig. 2f) in both flagellins of the ASU. This suggests that there is likely a mixture of FlaA and FlaB in the flagellar filament segments of our images. The fact that FlaA is the primary component of the filaments is supported by the strongly reduced motility of a *flaA* mutant strain due to severely truncated flagellar filaments^45^.

### Outer domain dimerization of the EHEC H7 flagellar filament

**Fig. 3:**
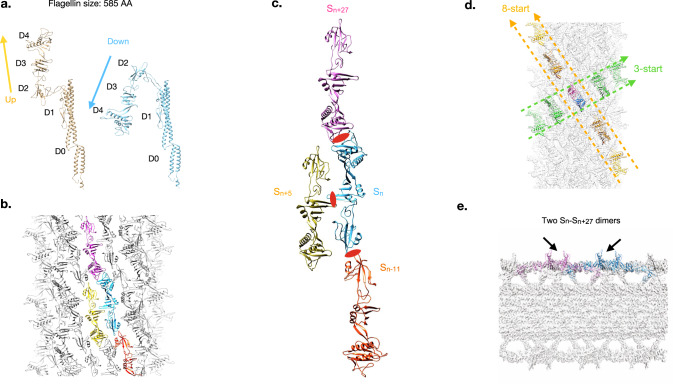
Structural details of the dimeric EHEC O157:H7 flagellar filament. **a** The two flagellin conformations of the EHEC H7 flagellar filament asymmetric unit. **b** Mesh-like sheath generated by interactions between four flagellin outer domains (blue, pink, yellow, and orange). **c** The EHEC H7 outer domains from subunit S_n_ interact with the outer domains of flagellins that are 5 (S_n + 5_), 11 (S_n + 11_), and 27 (S_n + 27_) subunits away. **d** The S_n_:S_n + 27_ dimer generates prominent right-handed 3-start and left-handed 8-start helices in the sheath surrounding the core. **e** View of the EHEC H7 flagellar filament cut halfway through the filament. The arrows are pointing to two S_n_:S_n + 27_ dimers, one blue and the other magenta, which are at the same radius.

Similar to the *S. meliloti* flagellar filament, the EHEC O157:H7 flagellar filament has a dimer symmetry (Table 1) with two flagellins in the ASU, one with an up and one with a down outer domain conformation (Fig. 3a). The much larger EHEC H7 outer domains (D2, D3, and D4) interact with outer domains from three other subunits forming a mesh-like ODS (Fig. 3b). In addition to forming dimers with outer domains that are five and 11 subunits away, every EHEC H7 outer domain also pairs with an outer domain that is 27 subunits away (Fig. 3c). This long-range S_n_:S_n + 27_ dimer results in an observable 8-start helix on the surface of the sheathed filament in addition to a 3-start (Fig. 3d and Supplementary Fig. 5b).

### Outer domain tetramerization of the *Achromobacter* flagellar filament

**Fig. 4:**
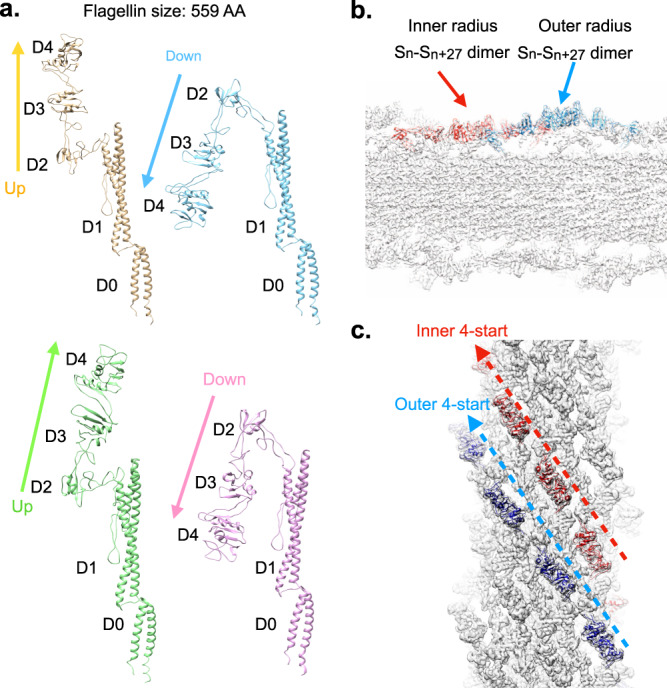
Structural details of the tetrameric *Achromobacter* flagellar filament. **a** The four flagellin conformations within the *Achromobacter* flagellar filament asymmetric unit. **b** *Achromobacter* outer domain interactions from subunits S_n_:S_n + 27_ occur at two different radii. **c** The two different dimer conformations result in two left-handed 4-start helices on the surface of the *Achromobacter* flagellar filament.

Like the EHEC H7, the *Achromobacter* ODS is also formed by interactions between flagellins that are five, 11, and 27 subunits away (Fig. 3c). However, there are four flagellins in the *Achromobacter* ASU, with two up and two down conformations (Fig. 4a). The key distinction is that all of the EHEC H7 S_n_:S_n+27_ dimers form at a single radius (Fig. 3e) while the *Achromobacter* S_n_:S_n+27_ dimers occur at two different radii (Fig. 4b). Consequently, the *Achromobacter* outer domain exists in four conformations and the filament has four flagellins in the ASU with an axial rise and twist for the outer domains that is four times larger than that of the flagellar core (Table 1). This tetrameric symmetry is characterized by two 4-start helices that occur at different radii (Fig. 4c). This can be viewed as a reduction of the 8-start helix observed in the EHEC H7 outer domains (Fig. 3d and Supplementary Fig. 5b) to two 4-start helices occurring at different radii (Fig. 4c and Supplementary Fig. 5c).

### Outer domain tetramerization of the EPEC H6 filament results in a seam

**Fig. 5:**
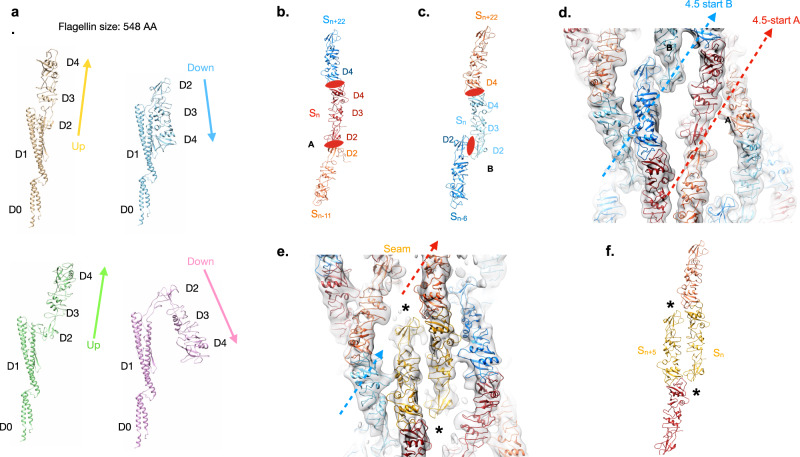
Structural details of the EPEC O127:H6 flagellar filament with a seam. **a** The four main flagellin conformations of the EPEC H6 flagellar filament. **b** An EPEC H6 outer domain dimer is generated by a symmetrical interaction between outer domain D2 from flagellin S_n_ with domain D2 of a flagellin 11 subunits away, S_n + 11_. This is named “D2 dimer A”. Flagellin outer domains also form a symmetrical dimer between domain D4 in subunit S_n_ with domain D4 from a flagellin 22 subunits away (S_n + 22_). **c** The second EPEC H6 outer domain dimer, “D2 dimer B”, is created between S_n_ and a flagellin six subunits away S_n−6_. **d** Surface view of the EPEC H6 flagellar filament showing the fit of the outer domain models into the low-resolution map. The two dimer conformations are indicated by “A” and “B” and form two right-handed 4.5-start helices. **e** A surface view of the EPEC H6 flagellar filament, rotated by 180° around the filament axis from the view in (**d**), showing the seam, *, with the subunits forming the seam in gold. **f** Interactions between outer domain subunits (gold) along the seam. The two subunits in the seam are 5 subunits away from each other in the filament (S_n_:S_n+5_).

The power spectrum of the EPEC O127:H6 flagellar filament (Supplementary Fig. 1e) superficially looks similar to the tetrameric *Achromobacter* filament. However, the Bessel order of many of the layer lines must be different because the EPEC H6 outer domains cannot be reconstructed using the tetrameric flagella symmetry of *Achromobacter*. We have determined that this is a result of a seam. The EPEC H6 filament ODS is created by an outer domain tetramer with most flagellins being in one of four flagellin outer domain conformations (Fig. 5a), but the details of this tetramerization are quite different from those in *Achromobacter*. Due to the presence of the seam, most but not all D2 domains are in one of these four conformations. In one dimer conformation, “D2 Dimer A”, domain D2 of one subunit forms a dimer with D2 from another flagellin that is 11 subunits away (Fig. 5b). In the other conformation, “D2 Dimer B”, flagellins that are 6-subunits away (S_n_:S_n + 6_) form dimers with each other (Fig. 5c). These two dimer conformations are easily observed in the 4.0 Å EPEC H6 map (Supplementary Fig. 7). While a large region of the ODS has very poor density, the resolution of another portion allows the creation of an ab initio atomic model of the complete flagellin structure. A 6.7 Å resolution volume was reconstructed asymmetrically where all flagellin subunits could be fitted into the density (Fig. 5d, e). Analysis of this low-resolution structure revealed the seam, which involves the lack of D2 dimer formation between two flagellin subunits (Fig. 5e). This seam is a consequence of a reduction of symmetry along the 9-start helices of a flagellar filament composed of dimeric flagellin such as EHEC H7. This results in two 4.5-start helices (Fig. 5d) that are discontinuous along a line indicated by the symbol “*” (Fig. 5e). The two outer domain subunits in the seam still form S_n_:S_n + 22_ dimers in domain D4. However, rather than forming an S_n_:S_n + 6_ or S_n_:S_n + 11_ dimer in domain D2, the two-seam subunits form S_n_:S_n + 5_ dimers with each other (Fig. 5f) similar to those forming the EHEC H7 and the *Achromobacter* ODSs.

### Disruption of the EHEC H7 ODS-forming dimer in domain D4 impairs motility

**Fig. 6:**
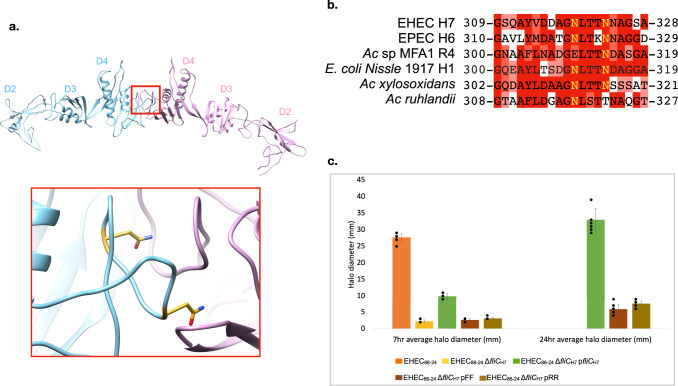
Mutagenesis of the EHEC H7 outer domain S_n_:S_n+27_ dimer reduces motility. **a** The S_n_:S_n + 27_ dimer is shown with the same color scheme as Fig. 2e. The inset reveals a close-up view of the dimer site. Residues that were mutated are shown in gold in the cyan subunit. **b** Multiple sequence alignment of the D4 dimer site for EHEC H7, EPEC H6, *Achromobacter* sp. MFA1 R4, *E. coli* Nissle 1917, *Achromobacter xylosoxidans* and *Achromobacter ruhlandii* flagellins. The two asparagine residues that were mutated in EHEC H7 (gold) are conserved with the exception of a glutamate residue in the second position in the *Achromobacter* flagellin. Sequences are colored by the conservation of chemical properties. **c** Soft agar (0.3 %) motility assay for EHEC 86–24 strains after 7 and 24 h. Data for the wild type (EHEC_86–24_) and wildtype with *fliC* deletion (EHEC_86–24_ Δ*fliC*_H7_) are not shown for the 24-h as the EHEC_86–24_ covered the entire plate and EHEC_86–24_ Δ*fliC*_H7_ showed no change. The pGEN-*fliC* strain complements the Δ*fliC* deletion strain. The FF and RR mutants were generated with the pGEN-*fliC* strain. Each bar represents the average halo diameter on the plate for each condition. A total of nine soft agar plates was measured for each condition. The black dots overlayed over each graph represent the data individual data points from which the bar graph was made from. The error bars represent the standard deviation for each condition.

The surface of the screw-like flagellar filament of *S. meliloti* and related species is thought to be an adaptation to swimming in highly viscous environments^46,47^, while the function of the ODS is unknown. To investigate ODS function, we aimed to disrupt its formation by mutation of several key residues in the EHEC H7 S_n_:S_n + 27_ domain D4 dimer interface (Fig. 6a). Examination of the amino acid sequences of the prominent S_n_:S_n + 27_ and S_n_:S_n + 22_ D4 dimer interfaces of the EHEC H7, *Achromobacter*, and EPEC H6 flagellins revealed a highly conserved sequence (Fig. 6b). Residues N319 and N323 in the EHEC H7 flagellin were chosen for mutagenesis (Fig. 6a, b). Two double mutants were constructed: N319F N323F (FF) and N319R N323R (RR) and mutant flagellar filaments isolated. Cryo-electron micrographs revealed that the FF mutant filament (Supplementary Fig. 8a) had a disrupted surface compared to that of wild-type EHEC H7 (Supplementary Fig. 8b). This disruption was also apparent when comparing the low-resolution FF reconstruction (Supplementary Fig. 8c) to the low pass-filtered wild-type H7 structure (Supplementary Fig. 8d). These differences between wild-type and mutant FF flagellar filaments were apparent in 2D class averages from Relion, where the wild-type H7 filaments displayed a characteristic 30 Å spacing corresponding to the D4 S_n_:S_n+27_ dimer (Supplementary Fig. 9a) while the FF mutant filaments did not have this spacing (Supplementary Fig. 9b).

Soft agar (0.3%) motility assays revealed drastically reduced motility of cells with the FF and RR mutant flagellar filaments as compared to the wild-type H7 (Fig. 6c). To ensure that the impaired motility of the mutants is not the result of altered filament production, we performed western blot analysis on motile bacteria and culture supernatants. We detected comparable levels of flagellin in washed cell fractions of the wild type and the deletion strains with complementation plasmids (Supplementary Fig. 10). Very low levels of flagellin were noticed in the culture supernatants and wash fractions after removing the cells by centrifugation indicating that non-functional flagellin monomers are not being exported directly into the cell milieu and that mutant filaments are not more fragile than wild-type filaments. We also examined wild-type and mutant FF EHEC cells by negative stain TEM (Supplementary Fig. 11) and confirmed that flagellar filaments from both strains were similar in length.

### ODS flagellar filaments demonstrate non-canonical polymorphic transitions

**Fig. 7:**
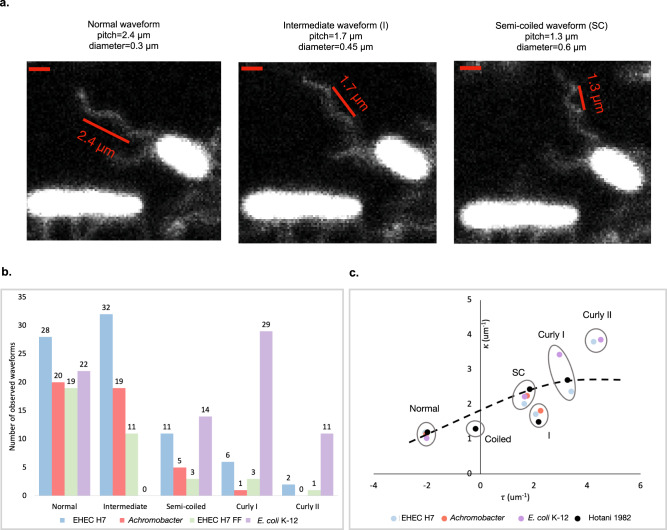
ODS flagellar filaments commonly adopt an “intermediate” waveform. **a** Images of fluorescently labeled *Achromobacter* sp. MFA1 R4 flagellar filaments with the three most common waveforms. Scale bars in the top left of each image correspond to ~1 µm. **b** Bar graph of waveforms observed in rotating EHEC H7, *Achromobacter* sp. MFA1 R4, EHEC H7 FF mutant, and *E. coli* K-12 flagellar filaments. There were 55, 34, 25, and 54 unique filaments whose polymorphic transitions were analyzed for the EHEC H7, *Achromobacter* sp. MFA1 R4, EHEC H7 FF mutant, and *E. coli* K-12 samples respectively. **c** Plot of curvature, κ, versus twist, τ, for the observed flagellar waveforms. The black dots are measured waveforms from Hotani^49^. The black dashed line is the predicted curvature κ^12^ as a function of τ. “I” stands for intermediate waveform and also includes the previously characterized “medium” waveform. “SC” stands for semi-coiled.

We used fluorescence microscopy to visualize labeled flagellar filaments on swimming wild-type EHEC H7 and FF mutant cells. Wild-type EHEC H7 cells exhibited different swimming behaviors depending on the number of flagellar filaments. Cells with one to two flagellar filaments had a tendency to be in a constant state of transitioning between waveforms and tumbled frequently (Supplementary Movie 1), while cells with more than two flagellar filaments exhibited long periods of straight swimming followed by robust tumbling or “double tumbling” where the filaments seem to take an extended period of time to return to the bundle causing the occurrence of two tumbles in a relatively short period of time (Supplementary Movie 2). Motile FF mutant cells entered periods of prolonged tumbling where the filaments exhibited an increased frequency of polymorphic switching (Supplementary Movie 3). Flagella from labeled wild-type EHEC H7, EHEC FF, and *Achromobacter* sp. MFA1 R4 frequently transitioned between the canonical normal and semi-coiled waveforms (Fig. 7a, Supplementary Movie 1, Supplementary Fig. 12). These same flagellar filaments also adopted a non-canonical intermediate waveform (Fig. 7a and Supplementary Fig. 12). The *Achromobacter* flagellar filaments appeared to primarily transition between these three waveforms as well as the semi-coiled form. The EHEC flagellar filaments also adopted the curly waveforms (Fig. 7b and Supplementary Fig. 12d). Images of fluorescently labeled, *E. coli* K-12 AW405 flagellar filaments, which do not possess an ODS, exhibited all the canonical flagellar waveforms as previously described^5,48^, and none of these filaments ever adopted the intermediate waveform (Fig. 7b and Supplementary Fig. 13).

The helical parameters of the intermediate waveform closely resembled those of the medium and unstable waveform in *Salmonella* flagella that was induced by mechanical force^49^. The plot in Fig. 7c presents filament curvature, κ, versus twist, τ, for each of the observed filament waveforms from wild-type EHEC O157:H7, *Achromobacter*, and *E. coli* K-12 AW405 compared to published values^12,49^. The parameters used to calculate this plot are listed in Supplementary Table 2. A notable difference was the greater stability of the intermediate waveform in *Achromobacter* and wild-type EHEC H7 filaments (Supplementary Movie 4 and 5) compared to the FF mutant filaments, which underwent frequent polymorphisms. These frequent transitions suggest an inherent instability of the disrupted dimer interface (Supplementary Movie 6).

### The ODS prolongs *E. coli* tumbling

**Fig. 8:**
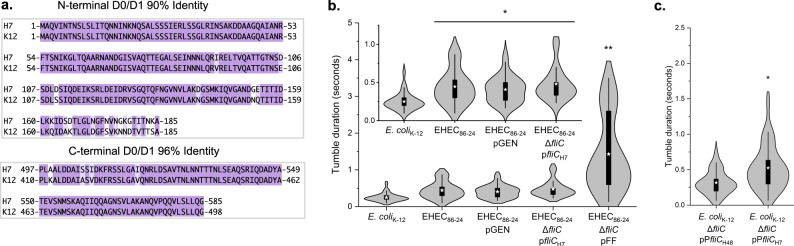
The H7 ODS prolongs *E. coli* tumbling. **a** Alignment of EHEC H7 and *E. coli* K-12 sequences in domains D0 and D1. Color indicates the conservation of chemical properties. **b** Tumbling times for various strains of *E. coli*. The strains are *E. coli* K-12, WT EHEC O157:H7 (EHEC_86–24_), WT EHEC O157:H7 with empty pGEN vector (EHEC_86–24_ pGEN), EHEC O157 Δ*fliC* complemented with the H7 *fliC* in the pGEN vector (EHEC_86–24_ Δ*fliC* pFliC_H7_), and the EHEC H7 FF mutant (EHEC_86–24_ Δ*fliC* pFF) are shown. The inset shows the tumbling times for all strains except the FF mutant. At least 50 cells were analyzed for each measurement. Asterisks represent statistically significant differences of the given strain(s) from other strain(s) (*P < 0.01; **P < 10^−6^). The *p*-values for EHEC_86–24_, EHEC_86–24_ pGEN, and EHEC_86–24_ Δ*fliC* pFliC_H7_ compared to *E. coli* K-12 are 0.000106, 0.000105, and 0.000006 respectively. The *p*-value for EHEC_86–24_ Δ*fliC* pFF compared to *E. coli* K-12 is 0.000000735. Each strain left to right the maximum (max) and minimum (min) tumbling times were 0.63 s max and 0.1 s min for *E. coli* K-12, 0.93 s max and 0.1 s min for EHEC_86–24_, 0.87 s max and 0.17 s min for EHEC_86–24_ pGEN, 1.1 s max and 0.23 s min for EHEC_86–24_ Δ*fliC* pFliC_H7_. **c** Tumbling times for *E. coli* K-12 HCB5 (Δ*fliC*) cells with the non-sheathed H48 flagellar filament (*E. coli* K-12 Δ*fliC* pP*fliC*_H48_) and the sheathed H7 flagellar filament (*E. coli* K-12 Δ*fliC* pP*fliC*_H7_). Asterisks represent statistically significant differences of the indicated strain(s) from other strain(s) (*P < 0.003). The exact *p*-value is 0.001845. For 8b and 8c significance was determined using a two-tailed *t*-test assuming two samples with unequal variance. Tumbling events for 30 unique cells over three independent experiments were measured for each condition. The boxes in the violin plots represent the interquartile range. The whiskers of the box plot correspond to the outer most data points that is within 1.5 multiplied by the interquartile range.

To compare the run-and-tumble motility behavior of the various EHEC strains to that of *E. coli* K-12 AW405 (K-12), we employed phase-contrast microscopy and recorded movies of swimming cells at 30 frames per second. The D0 and D1 domains of the *E. coli* K-12 flagellin exhibit over 90% sequence identity to the H7 flagellin (Fig. 8a). However, its outer domains show no significant sequence homology to H6 or H7 and do not form a sheath^50^. In contrast, the K-12 outer domains align well and have 35% sequence identity with the *S. typhimurium* FliC outer domains (Supplementary Fig. 14), a likely indicator of a conserved fold. Indeed, structure prediction of the *E. coli* K-12 outer domains using AlphaFold^51^ results in a model with a fold quite similar to the *S. typhimurium* flagellin outer domains and no structural similarity to the EHEC H7 flagellin (Supplementary Fig. 15). *E. coli* K-12 cells exhibited the highest average swimming velocity at about 25 µm/s (Table 2), which agrees with previous published values^52,53^. Cells frequently displayed extended periods of straight swimming followed by very short tumbles with a duration of ~220 ms (Fig. 8b and Supplementary Movie 7). EHEC cells expressing the wild-type H7 flagellar filament either from its native locus or from a plasmid had swimming velocities of about 19 µm/s (Table 2). Interestingly, these cells exhibited prolonged tumbles of around 500 ms, twice as long as that of K-12 wild type (Fig. 8b and Supplementary Movie 8). EHEC strains with the FF mutant swam more slowly (Table 2) and stayed in the tumble mode for ~1500 ms (Fig. 8b and Supplementary Movie 9).

**Table 2:** Swimming velocity measurements for *E. coli* K-12 and EHEC strains.

Strain	Velocity (µm/s)
*E. coli*_K-12_	25.2 ± 0.2
*E. coli*_K-12_ ∆*fliC* pP*fliC*_H48_	19.9 ± 0.7
*E. coli*_K-12_ ∆*fliC* pP*fliC*_H7_	19.0 ± 0.1
EHEC_86–24_ pGEN	19.5 ± 2.4
EHEC_86–24_ Δ*fliC* p*fliC*_H7_	19.0 ± 0.1
EHEC_86–24_ Δ*fliC* pFF	13.8 ± 1.5
EHEC_86–24_ Δ*fliC* pRR	13.2 ± 1.7

For each strain 50 cells were analyzed.

To investigate whether the H7 flagellar filament is the cause of the prolonged tumble duration, we complemented the *E. coli* K-12 *fliC* deletion strain HCB5 with EHEC *fliC* (FliC_H7_) and the native K-12 *fliC* (FliC_H48_) from plasmid pTrc99a using the native *fliC* promoters (pP*fliC*_H48_ and pP*fliC*_H7_). The *E. coli* K-12 ∆*fliC* strain regained swimming motility when complemented with either EHEC or K-12 *fliC* (Table 2). Interestingly, complementation with pP*fliC*_H7_ resulted in tumbling durations similar to the wild-type EHEC H7 strains with ~500 ms and nearly twice of the *E. coli* K-12 strain AW405 (Fig. 8c). These results suggest that the H7 flagellin is the cause of extended tumbles.

## Discussion

Each of the four presented flagellar filament structures exhibits a different level of structural complexity: The *S. meliloti* and EHEC H7 structures are formed with outer domain dimers, while the *Achromobacter* and EPEC H6 structures are produced by outer domain tetramers. The effects of dimerization and tetramerization of flagellin outer domains have been seen before in diffraction patterns of flagellar filaments, but these studies were unable to determine the nature of the dimerization or tetramerization^35,41,54^. Flagellar filaments without a seam were defined as helically perturbed, and ones with a seam as non-helically perturbed. In the light of our findings, the use of the term perturbation should be re-evaluated, because the generation of these structures involves a full 180° rotation of every other flagellin’s outer domain. In addition, the ODS we describe in this paper should not be confused with other flagellar sheaths which are either membranous^55^ or created by proteins other than flagellin^56^.

A structural homology search of the individual flagellin outer domains from each structure using the Dali server^57^ yielded only a few potential structural homologs in unrelated proteins (Supplementary Table 3). However, none of these hits were convincing in terms of sharing an overall fold. Since the outer domains of the four structures formed dimers about a two-fold axis in strikingly similar manners (Supplementary Fig. 3), this led us to wonder if there was any commonality between the *S. meliloti* dimer site and that of the three ODS flagellar filaments’ D4 dimer site. Interestingly, the electrostatic potential of the EHEC H7 and *Achromobacter* D4 dimers along with the *S. meliloti* D2 dimer all have a negative charge at the dimer site (Supplementary Fig. 16a). In the case of the EHEC H7 dimer one aspartate at position 316 from each subunit is at the interface (Supplementary Fig. 16b) while for *S. meliloti* the negative charge is due to glutamates from each subunit (Supplementary Fig. 16c). It has been suggested that divalent cations are critical to the stability of the *S. meliloti* outer domains. We found no evidence of cation density being preset in these negatively charged pockets in any of these structures, but this does not necessarily exclude the possibility. Another possibility is that the dimer interactions are strengthened at lower pH values is a remote possibility that the EHEC H7 dimer site functions as some sort of aspartic protease, but it is missing the classic Asp-Thr-Gly motif found in most aspartic proteases^58^. The discovery of proteolytic flagellin outer domains^32^ might give some credibility to this possibility. The HIV-1 aspartic protease dimer^59^ (Supplementary Fig. 16d) bears some similarity to the negatively charged dimer sites in our models due to the interfacing aspartate residues from both subunits of the protease dimer.

The surface areas of the *S. meliloti*, EHEC H7, and *Achromobacter* Sn:Sn_+5_ and Sn:Sn_+11/−11_ interfaces were calculated using PISA^60^. The values for these three structures are all comparable to each other and reveal increased contacts between the S_n_:S_n+5_ and S_n_:S_n+11/−11_ compared to *B. subtilis* and *S. typhimurium* flagellar filaments (Supplementary Table 4), which is due to the dimers that form the screw-like surfaces and ODSs. Interestingly, the Sn:Sn_+11/−11_ contacts are much less extensive than those of the *C. jejuni* flagellar filament, which outer domains make extensive contacts along the same protofilament to compensate for its destabilized toll-like receptor 5 (TLR5) sequence^16^. Contacts made between adjacent *C. jejuni* flagellin outer domains might serve an additional role in stabilizing the unique motility associated with the two polar flagellar filaments of *C. jejuni*, one of which wraps itself around the cell body likely allowing for better penetration of host mucus^61^. The outer domain interactions of *S. meliloti*, EHEC H7, *Achromobacter*, and EPEC H6 flagellins are unique from all other high-resolution flagellar filament structures because they form dimers or tetramers on the surface of the filament, which generate different helical lattices around their flagellar cores. These new outer domain interactions then alter the polymorphism of the flagellar filament in the form of the constricted normal waveform for the *S. meliloti* screw-like flagellar filament^62^ and the intermediate waveform for flagella with ODSs.

The intermediate waveform with an average pitch of 1.8 µm and diameter of 0.45 µm adopted by the ODS flagellar filaments is strikingly similar to the previously discovered unstable right-handed “medium” waveform with a pitch of 1.9 µm and diameter of 0.43 µm^49^. This medium waveform required the same magnitude of torque for the transition to curly or semi-coiled forms, while the torque needed for the transition from medium to normal was very small. Mutations in the EHEC H7 S_n_:S_n + 27_ dimer site result in increased transitions between the normal and intermediate waveforms. Although FF mutant flagella still form the intermediate waveform, we believe that it is destabilized in a similar manner to the medium waveform^49^. The wild-type S_n_:S_n + 27_ dimer site could stabilize the intermediate waveform, thus increasing the torque required for the intermediate-to-normal transition.

The ODS and the intermediate waveform of the H7 filament might prolong tumbling time by creating an additional step in the canonical run-and-tumble scheme^6^. Upon switching to CW rotation, the EHEC H7 flagellar filament might first transition from normal to intermediate, then to semi-coiled, and finally to curly. Transitioning through an additional waveform might prolong the time spent in tumbling mode. Incomplete polymorphic transitions also occur during run-and-tumble motility^5,6,63^. We speculate that the additional intermediate waveform might not prolong tumbling in incomplete transitions when the motor rotation reverses back to CCW before the transition to curly^6^. This is consistent with our observation that cells with the H7 flagellar filament undergo quick tumbles similar to cells with the H48 filament. We observed fewer EHEC H7 and *Achromobacter* flagellar filaments that adopted the curly waveform, which could suggest that the curly waveforms are disfavored in ODS filaments.

The intermediate waveform of our ODS flagellar filaments is likely similar to the constricted normal waveform of flagellar filaments with screw-like surfaces^62,64^, because both waveforms have shorter pitches and diameters than the normal waveform and are distinguishable from the semi-coiled and curly waveforms. The S_n_:S_n+5,_ S_n_:S_n+6_, and S_n_:S_n+11/−11_ dimers and the 3-start helix formed on the surface likely allow for the formation of these unique shorter-pitch waveforms. However, without the additional S_n_:S_n+27_ or S_n_:S_n+22_ dimer, such waveforms might be rather unstable in *E. coli* cells with bidirectional flagellar rotation that require flagellar polymorphism. A different behavior is observed for the unidirectional flagellar rotation in *S. meliloti*, which does not require a change in flagellar waveform during run-tumble transitions^62^. The S_n_:S_n + 27_ dimers of the EHEC H7 and *Achromobacter* flagellar filaments and the S_n_:S_n + 22_ dimers of the EPEC H6 flagellar filament may stabilize normal and intermediate waveforms. A weakened interaction in the EHEC H7 FF mutant flagellar filament with the disrupted S_n_:S_n + 27_ dimer site would explain why these mutant cells had drastically worse motility and seemed to constantly transition between waveforms.

Pathogens such as EHEC O157:H7 and EPEC O127:H6 must be able to swim through the intestinal mucus layers to reach the epithelium where they ultimately form attaching and effacing lesions^65–69^. The genus *Achromobacter* is found in water and soil^70^ the latter which has a relatively high viscosity. Some *Achromobacter* species such as *A. xylosoxidans* and *A. ruhlandii* were also characterized as opportunistic pathogens in cystic fibrosis patients^71^. These two opportunistic pathogens have flagellins with high sequence homology in their outer domains with the ODS flagellins characterized in this manuscript and have the conserved S_n_:S_n + 27_ or S_n_:S_n + 22_ dimer interface (Fig. 4b). The common attribute between bacteria that produce these screw-like and ODS flagellar filaments is that they are all motile in environments that have higher viscosities than water. The mucosal environment is quite heterogenous and some pathogens prefer to swim through “gaps” in the mucus layers to reach the epithelium^72^. It has been shown that *E. coli* K-12 cells in porous environments exhibit long periods of being trapped followed by “hopping”^73^. The increased tumbling mediated by the outer domains of non-canonical flagellar filaments may allow for better reorientation in viscous and porous environments during a tumble, preventing the bacteria from being trapped while traversing these environments. These screw-like and ODS flagellar filaments are just one strategy that bacteria may use to cope with high-viscosity environments as there are many bacteria with canonical flagellar filaments that inhabit these environments. The outer domains of canonical flagellar filaments likely provide other advantages in environments with higher viscosity. In *S. typhimurium*, for example, methylation of both phase-1 and phase 2 flagellins are implicated in adhesion and invasion into host cells^27^.

In addition to their impact on motility, it has been shown that EHEC H7 and EPEC H6 ODS flagellar filaments function as adhesins^25,26^, and ODS-forming H1, H6, and H7 flagellin monomers trigger increased TLR5 activation^28,29,74^. The flagellar filament-mediated adhesion seems to be a general property of canonical and ODS flagellar filament outer domains as several studies show similar adhesive capabilities of both *S. typhimurium* and EHEC H7 flagellar filaments to the same surfaces^75^. The dimerization of the flagellin outer domains could also explain the increased TLR5 activation seen by the ODS-forming H1, H6, and H7 *E. coli* flagellins when they are bound to TLR5^28,29,74^. A crystal structure of the *S. typhimurium* flagellin FliC bound to TLR5 revealed a complex consisting of two TLR5:flagellin heterodimers^76^. where domains D1 are bound by TLR5 and domains D2 of each flagellin are in close proximity (Supplementary Fig. 17). For ODS-forming EHEC H7 and EPEC H6 flagellins these complexes could be stabilized by dimerization between the flagellin outer domains in the complex allowing for more robust TLR5-signaling. The outer domain dimers might also stabilize the flagellin subunits in detached flagellar filaments hindering the dissociation of the filament into monomers. A lower concentration of flagellin monomers might be advantageous during later stages as detached ODS flagellar filaments could possibly result in lower levels of TLR5 activation than canonical flagellar filaments.

Flagellin can be the single most abundant protein produced by bacteria and is thus under intense selection^77^. An example of such selection is the Macnab experiment: in stirred liquid culture, where flagellar motility provides no advantage, spontaneous mutations in flagellar genes lead to the complete loss of flagellar filament in only 10 days, because bacteria that fail to synthesize flagellin have a slightly increased fitness^78^. The outer domains required to produce ODS flagellar filaments are 50–100 amino acid residues larger than the *E. coli* K-12 and *S. typhimurium* flagellar filaments. Thus, producing these flagellar filaments with ODS would be even more energetically costly than producing flagellar filaments without a sheath. Given the intense selective pressure on their synthesis, flagellin oligomerization and the subsequent effect on motility may be viewed as a mechanism for specific adaptations to environmental niches.

## Methods

### Generation of EHEC H7 flagellin mutants and EHEC and K-12 complementation plasmids

The H7 *fliC* gene was deleted from EHEC 86–24 using lambda Red recombination as published^79^. *fliC* was cloned into the pGEN-MCS vector using restriction digestion (NheI and HindIII) and ligation. Potential mutations were chosen based on changes in predicted stability calculated by the FoldX^80^ and BindProfX^81^ programs using the EHEC H7 flagellin outer domain structure in both monomer and dimer forms as input. Mutations were made in the pGEN-*fliC* vector using the Q5 mutagenesis kit according to the manufacturer’s instructions.

For *fliC* complementation using native *fliC* promoters, *fliC* genes were amplified from genomic DNA using a primer pair that included a 280-bp region upstream of the *fliC* translation start site to generate 2050 bp (EHEC) and 1789 bp (K-12) fragments, respectively. Fragments were digested and cloned into the EcoRI and SalI sites of the pTrc99a plasmid.

Deletion, mutations, and complementation plasmids were all verified by Sanger sequencing.

### EHEC O157:H7 soft agar motility assays

EHEC strain 86–24 was grown aerobically in LB overnight at 37 °C with shaking. The next day, cultures were diluted 1:100 into fresh LB and grown to mid-exponential phase (OD_600_ 0.4–0.6). From these, a 1 µL aliquot of culture was stab inoculated into motility plates (LB with 0.3% agar). Plates were incubated for 7–24 h at 37 °C and halo diameter was measured.

### Phase-contrast swimming behavior assays

EHEC strains were diluted from overnight LB broth cultures into fresh media with appropriate antibiotics and incubated at 37 °C in a roller drum to an OD_600_ of ~1.5. *E. coli* K-12 strains were incubated in tryptone broth at 37 °C in a roller drum to an OD_600_ of ~0.5. Cells were pelleted in round-bottom tubes for six minutes at 3000 × *g*, the spent media removed, and cells were suspended in an equal volume of motility buffer (10 mM potassium phosphate, 10 mM lactate, and 70 mM sodium chloride, pH 7.0) in a roller drum at 20 rpm for ~20 min. The centrifugation and suspension steps were repeated once prior to visualization Videos were recorded at 30 fps using a Nikon Eclipse E600 phase-contrast microscope equipped with a custom Nikon camera from Imaging Source. Swimming velocities were quantified using TumbleScore scripts written in MATLAB^82^. Tumble durations were measured manually using frame-by-frame playback of videos. Reported values are averages and standard deviations of at least 30 independent tumbling events.

### Western blotting

Bacteria were grown and washed as described for swimming velocity and tumbling analyses. At each stage of the washing procedure, 10 µl of the corresponding sample was mixed with an equal volume of Laemmli buffer containing β-mercaptoethanol and samples were boiled for ten minutes prior to tank blot transfer as previously described^83^. Blots were incubated in blocking buffer consisting of PBS with 5% milk for at least one hour at room temperature. Anti-FliC polyclonal antibody serum and horseradish peroxidase-linked goat anti-rabbit antibody were used at 1:10,000. Chemiluminescence signals were detected on ECL Hyperfilm.

### Fluorescence labeling of flagellar filaments

Flagellar filaments were labeled using established methods^5^ using the Alexa Fluor^TM^ 546 NHS Ester Protein Labeling Kit from ThermoFisher Scientific. Overnight cultures of EHEC H7 and *Achromobacter* sp. MFA1 R4 cells were checked for motility using the DIC mode on Zeiss LSM 880. These cells were then pelleted at low centrifugation speeds of 1.2k × *g* for 5 min and washed three times by resuspension in 1× PBS and pelleting at the same low speed for 5 min. The final suspension volume of cells in 1× PBS was 500 µL and 50 uL of 1 M bicarbonate was added to the suspension. This mixture was then transferred to the tube containing the AlexaFluor probe and rotated for 1 h in the dark at room temperature. For *E. coli* K-12 AW405 cells the steps were exactly the same however cells were grown to OD of 0.6 prior to labeling.

### Fluorescence confocal microscopy

Fluorescently labeled cells were imaged using a laser scanning confocal microscope (Zeiss LSM 880) with a 40x water immersion objective (N.A. 1.2, Zeiss). The sample was excited at 561 nm and the emission was collected between 566 and 679 nm. For *E. coli* K-12 and *Achromobacter* sp. MFA1 R4 cells images of 512 × 512 pixels (53.14 µm × 53.14 µm) were collected with intervals of 240 milliseconds taken between each frame. For the experiments with the WT EHEC H7 and FF mutants, images of 256 × 256 pixels encompassing a larger physical area (83.78 µm × 83.78 µm) were obtained with 210 milliseconds between each image. Just prior to imaging, cells were diluted into 1:50 LB with or without 10^−4^% tween.

### Flagellar filament preparation

H7 flagellar filaments were prepared as published^25^. EPEC ICC526 (EPEC O127:H6 Δ*bfpA* + Δ*espA*) was transformed with *flhDC* plasmid. To purify flagella, exponentially growing EPEC ICC526 culture (*OD*_600_ 0.6) carrying pflhDC was induced with 0.5 mM isopropyl β-D-1-thiogalactopyranoside (IPTG) for 3 h. Bacteria were then pelleted by centrifugation at 7000 × *g* and the pellet was suspended in 500 μl of cold, 1 M Tris/HCl, 100 mM NaCl buffer (pH 6.5). Bacteria were deflagellated by passing multiple times through a 25 G needle until viscosity decreased. The deflagellated cells were pelleted by centrifugation at 10,000 × *g* for 15 minutes. The flagella in the supernatant were centrifuged at 10,000 × *g* at 4 °C to remove small debris. The resulting pellet was suspended in 500 μl of buffer. Purity of the flagella was analyzed by SDS PAGE.

*Sinorhizobium meliloti* flagellar filament purification was done essentially as described^45^ with slight modifications. *S. meliloti* wild-type strain RU11/001 grown in TYC (0.5% tryptone, 0.3% yeast extract and 0.13% CaCl_2_ × 6 H_2_O) at 30 °C was diluted to an OD_600_ of 0.05 in RB (6.1 mM K_2_HPO_4_, 3.9 mM KH_2_PO_4_, 1 mM MgSO_4_, 1 mM (NH_4_)_2_SO_4_, 0.1 mM CaCl_2_, 0.1 mM NaCl, 0.01 mM Na_2_MoO_4_, 0.001 mM FeSO_4_, 2 µg/l biotin). Twenty Bromfield plates (0.5% tryptone, 0.3% yeast extract and 0.13% CaCl_2_ × 6 H_2_O) were over-laid with 10 mL of the diluted culture and grown for 15 h at 30 °C to an OD_600_ of 0.6. Cells were harvested by centrifugation for 8 min at 8000 × *g* and suspended in 100 mL motility buffer (0.5 mM CaCl_2_, 0,1 mM EDTA, 20 mM HEPES [pH7.2]). Flagella were sheared by agitation in a mixer at full speed for 20 seconds, separated from cells by centrifugation at 8000 × *g* for 7 min, 15,000 × *g* for 15 min, and 30,000 × *g* for 50 min at 4 °C. Purified flagella were sedimented at 87,000 × *g* for 2 h at 4 °C, washed once and suspended in 200 µL motility buffer. Purity of the flagella was analyzed by SDS PAGE.

### Cryo-EM preparation

Flagella samples were prepared for cryoEM using established protocols^13,16^. Plunge freezing was done using a Vitrobot^TM^ Mark II plunge freezer. Briefly, 3–4.5 µL of flagellar filament sample was applied to a lacey carbon grid. The droplet was blotted for 3.5 s with the blot force settings ranging from 3 to 6 and then plunged into liquid ethane.

### Cryo-EM image acquisition

The image acquisition settings were the same as those previously published^16^, with data acquisition using either EPU (ThermoFisher Scientific) or cryoSPARC.

### Image processing and helical reconstruction

For the *Achromobacter* sp. MFA1 R4 and EHEC H7 flagellar filaments image processing and helical reconstruction were performed as published^13,16^ using the Spider^84^ and Relion 3^85^ software packages. Motion correction was performed using MotionCorr2^86^.

The *S. meliloti* and EPEC H6 image processing and structural determination were performed using cryoSPARC^87^. Images of *S. meliloti* and EPEC H6 flagellar filaments were motion corrected using the Patch Motion Correction process and contrast transfer function (CTF) estimation was done using Patch CTF Estimation. Initial subsets of flagellar filament segments (500–1000) were manually picked and underwent 2D classification. Selected class averages were then used as inputs for both Template Picker and Filament Tracer. More quality flagellar filament images were selected using the Template Tracer function then Filament Tracer. The desired minimum separation between particles was determined based on the expected axial rise of the helical symmetry to be used during reconstruction. For *S. meliloti* a featureless cylindrical volume was used as the starting volume for reconstruction and an initial resolution reached 4.0 Å, and the helical symmetry converged to that shown in Table 1. Local CTF Refinement followed by helical reconstruction of these CTF refined particles resulted in a final volume with 3.5 Å resolution using the FSC = 0.143 map:map criterion. For EPEC H6 the core domains were reconstructed with a monomeric symmetry and a resolution of ~4.5 Å was achieved. However, due to a seam in the outer domains, helical reconstruction could not reach high resolution for the full filament.

### Asymmetric reconstruction of the EPEC H6 flagellar filament

The 4.5 Å resolution EPEC H6 core domain volume from helical reconstruction was filtered to 25 Å resolution and used as an input into the cryoSparc Homogenous Refinement program. A curved reconstruction of the H6 flagellar filament was obtained with core domain resolution around 3.5 Å and outer domains around 6 Å from ~270,000 particles. 3D variability analysis was performed, and the output was clustered. The particles were then subjected to CTF refinement. Using local refinement and a masking out of the core domains we achieved 4.3 Å resolution (FSC = 0.143 map:map) for the EPEC H6 outer domains. A large portion of the outer domains on the inner most curved side of the reconstruction had very poor density likely due to misalignment of particles. Using cryoSPARC’s 3D Variability analysis and solving for three different modes of heterogeneity we obtained several clusters (particles and their corresponding volumes) with differences in the overall quality of the outer domains as well as curvature. A single cluster with ~60,000 particles showed low resolution (~8 Å) features corresponding to the outer domain dimers without the issue of non-uniform density in the inner curved region. Analysis of this region showed an apparent seam. Attempts to use the particles and volume from this 3D variability cluster for a reconstruction with extensive rotation and shift searches resulted in a final volume with no seam and very poor density in the corresponding inner curve. Local refinement of the reconstruction from these 60,000 particles starting from the cluster low-pass filtered to 12 Å and a mask around the outer domains resulted in a volume that retained the seam in the ODS with an estimated 6.3 Å resolution for the outer domains (FSC = 0.143 map:map).

### Model building

Model building for the flagellar filaments in this study was published for other flagellar filaments^13,88^. The corresponding density for an individual flagellin was isolated from the filament reconstruction using UCSF Chimera^89^. The D0/D1 model of an already deposited flagellin (PDB 5WJY) was fit into the corresponding region of each subunit’s density map and then the residues were mutated to the appropriate ones for each structure using Coot^90^. For the outer domains, the chains were traced manually in Coot and then refined using Rosetta CM^91^. Filament models were then generated in UCSF Chimera and refined and validated in Phenix^92^ using real-space refinement^93^ and real-space validation. For the H6 flagellar filament, a homology model was initially generated using Swiss-Model^94^. This was subsequently refined in coot and much of the model was manually rebuilt using coot and refined in phenix. For refinement of the H6 model to the high-resolution map, the model was fit into only the regions of good outer domain density in the map.

### *Achromobacter* sp. MFA1 R4 structural determination

The *Achromobacter* sp. flagellar filament was a contaminant of a pili prep from *Agrobacterium tumefaciens*. Surprisingly, cryoEM images of the *A. tumefaciens* preparation revealed no pili at all but two very different flagellar filaments: thin flagellar filaments ~140 Å in diameter (yellow triangle Fig. 1c) with a canonical flagella power spectrum (Supplementary Fig. 1a), and much larger flagellar filaments (~220 Å diameter) with ODS (red arrow Fig. 1c) and a tetrameric flagella power spectrum (Supplementary Fig. 1d). SDS-PAGE followed by silver staining was as published^95^ and identified numerous gel bands arising from many contaminants. Multiple bands were excised from the gel and submitted for mass spectrometry analysis at the University of Virginia Biomolecular Analysis Facility.

The gel pieces from the band were transferred to a siliconized tube and washed in 200 µL 50% methanol. The gel pieces were dehydrated in acetonitrile, rehydrated in 30 µL of 10 mM dithiothreitol (DTT) in 0.1 M ammonium bicarbonate, and reduced at room temperature for 0.5 h. The DTT solution was removed, and the sample was alkylated in 30 µL 50 mM iodoacetamide in 0.1 M ammonium bicarbonate at room temperature for 0.5 h. The reagent was removed, and the gel pieces were dehydrated in 100 µL acetonitrile. The acetonitrile was removed, and the gel pieces rehydrated in 100 µL 0.1 M ammonium bicarbonate. The pieces were dehydrated in 100 µL acetonitrile, the acetonitrile removed, and the pieces completely dried by vacuum centrifugation. The gel pieces were rehydrated in 20 ng/µL trypsin in 50 mM ammonium bicarbonate on ice for 30 min. Any excess enzyme solution was removed and 20 µL 50 mM ammonium bicarbonate was added. The sample was digested overnight at 37 ^o^C and the peptides were extracted from the polyacrylamide in a 100 µL aliquot of 50% acetonitrile/5% formic acid. This extract was evaporated to 9 µL for MS analysis.

The LC-MS system consisted of a Thermo Electron Q Exactive HF mass spectrometer with an Easy Spray ion source connected to a Thermo 75 µm × 15 cm C18 Easy Spray column. 1–3 µL of the extract was injected and the peptides eluted from the column by an acetonitrile/0.1 M formic acid gradient at a flow rate of 0.3 µL/min over 1.0 h. The nano spray ion source was operated at 1.9 kV. The digest was analyzed using the rapid switching capability of the instrument acquiring a full scan mass spectrum to determine peptide molecular weights followed by product ion spectra (10 HCD) to determine the amino acid sequence in sequential scans.

Mass spectrometry identified the presence of peptides belonging to *A. tumefaciens* flagellin as well as potential peptide hits for the flagellin from the sheathed flagellar filament from various strains of *Achromobacter*. A BLAST search^96^ of two peptide hits from the Mass Spec, FTANVRGLTQAAR, and ISEQTDFNGVK, identified only two proteins in UniproKB with 100% coverage in both sequences they were flagellin from *Achromobacter* sp. 2789STDY5608615 and *Achromobacter* sp. MFA1 R4. *Achromobacter* sp. 2789STDY5608615 was a partial sequence in the UniprotKB database missing a small part of its C-terminal D0 sequence and its outer domain sequence was about 15 amino acids too large, while the *Achromobacter* sp. MFA1 R4 was a better fit. Pure *Achromobacter* sp. MFA1 R4^38^ cells were then obtained, and the flagellar filaments were confirmed to be sheathed using negative stain TEM.

### Modeling of the *S. meliloti* flagellar filament

A sequence alignment of the four *S. meliloti* flagellins is shown in Supplementary Fig. 12. Both flagellin subunits in the ASU correspond to a flagellin that is ~395 amino acids in length which would exclude either subunit being FlaC. FlaA has Met300 which corresponds to Ala298 in FlaD. Both subunits in the ASU show a large side-chain density at that position (no dihedral symmetry has been imposed on the outer domains) which could correspond to a methionine but not alanine (Supplementary Fig. 13a). Thus, we can exclude FlaD from being one of the main components of the flagellar filament in our images. There are several regions where side-chain density corresponding to the FlaA sequence would be difficult to explain with the FlaB sequence, such as those corresponding to FlaA residues Glu205 and Asn294 which correspond to Ala205 and Gly294 in FlaB (Supplementary Fig. 13b, c). Given that the densities of the two subunits in the ASU are nearly identical, it seems unlikely that the screw-like surface is formed specifically by a FlaA:FlaB heterodimer and most of the dimers must be formed by FlaA:FlaA homodimers. But it is still possible that some population of the segments used for reconstruction contained FlaB:FlaB homodimers or even FlaA:FlaB heterodimers that degrade the side-chain density of residues like Glu205 and Asn294 when averaged with the predominant FlaA:FlaA dimers.

### AlphaFold predictions

The *E. coli* K-12 flagellin model was predicted using the AlphaFold^51^ option found in daily builds of UCSF ChimeraX^97^.

### Multiple sequence alignments

Figures for sequence alignment were made using Jalview-2^98^. All alignments were done using Clustal Omega^99^.

### Analysis of flagellar filament waveforms

Movies of fluorescently labeled bacteria with attached flagellar filaments were analyzed in Fiji^100^. Rotating flagellar filaments were analyzed for polymorphic transitions. Each unique transition was measured and used to calculate the parameters in Fig. 7c and Supplementary Table 2.

### Reporting summary

Further information on research design is available in the Nature Research Reporting Summary linked to this article.

## Supplementary information

Supplementary Information

Description of Additional Supplementary Files

Supplementary Movie 1

Supplementary Movie 2

Supplementary Movie 3

Supplementary Movie 4

Supplementary Movie 5

Supplementary Movie 6

Supplementary Movie 7

Supplementary Movie 8

Supplementary Movie 9

Reporting Summary

## Data Availability

The data that support this study are available from the corresponding authors upon reasonable request. All atomic models were deposited in the Protein Data Bank and all density maps were deposited in the Electron Microscopy Database. The *S. meliloti* map and model are available as EMD-25215 and PDB 7SN9 [10.2210/pdb7SN9/pdb]. The EHEC H7 full filament map and model are available as EMD-25211 and PDB 7SN4 [10.2210/pdb7SN4/pdb]. The EHEC H7 outer domain sheath with D1 symmetry applied is available as EMD-25388. The EHEC H7 FF mutant structure is available as EMD-25212. The EPEC H6 high-resolution map and model are available as EMD-25213 and PDB 7SN7 [10.2210/pdb7SN7/pdb]. The lower resolution EPEC H6 map is available as EMD-25386, while the model of the two-seam subunits is available as PDB 7SQJ [10.2210/pdb7SQJ/pdb]. The *Achromobacter* map and model are available as EMD-25382 and PDB 7SQD [10.2210/pdb7SQD/pdb]. Source data are provided with this paper.

## Source data

Source Data
