# Supplementary material for: Flagellin outer domain dimerization modulates motility in pathogenic and soil bacteria from viscous environments
Source: Nat Commun. 2022 Mar 17;13:1422. doi: 10.1038/s41467-022-29069-y (PMC8931119; doi:10.1038/s41467-022-29069-y)
Supplement: Supplementary file 1 — Supplementary Information [file 41467_2022_29069_MOESM1_ESM.pdf]

Supplementary Table 1. Cryo-EM and model refinement statistics.

	**EHEC O157:H7 Full Filament**	**EPEC O127:H6 High Resolution map**	**Achromobacter MFA1 R4**	**S. meliloti**	**EHEC O157:H7 D1 Symmetry Map**	**EPEC O127:H6 Low resolution map with seam**	**EHEC H7 FF mutant**
PDB	7SN4	7SN7	7SQD	7SN9	N/A	7SQJ	~
EMDB	25211	25213	25382	25215	25388	25386	25212
Resolution 0.143 FSC Map:Map (Å)	3.6	4.2	3.7	3.5	4.1	6.3	~
Resolution 0.378 FSC Model:Map (Å)	3.6	4	3.7	3.7	~	6.7	~
Ramachandran favored (%)	94.3	91.3	90.7	93.6	~	~	~
Ramachandran allowed (%)	5.7	8.7	9.1	6.4	~	~	~
Ramachandran Outliers (%)	0	0	0.2	0	~	~	~
Clash score	7.5	14.5	15.23	8.5	~	~	~
Molprobity score	1.8	2.18	2.22	1.88	~	~	~
Bond angles rmsd (°)	0.003	0.7	0.61	0.51	~	~	~
Bond length RMSD (Å)	0.52	0.003	0.003	0.002	~	~	~

Supplementary Table 2. Helical waveform parameters of fluorescently labeled EHEC H7, *Achromobacter*, and *E. coli* K12 flagellar filaments. Twist and curvature are represented by the τ and κ respectively. The helix angle is represented by θ.

Bacteria	Waveform	Pitch (μm)	Diameter (μm)	τ(μm-1)	κ (μm-1)	θ (°)
EHEC H7 Wt	Normal	2.3	0.4	-2.1	1.2	30.8
EHEC H7 Wt	Normal II	1.8	0.5	2.1	1.7	40.0
EHEC H7 Wt	Semi-coiled	1.5	0.6	1.6	2.0	50.9
EHEC H7 Wt	Curly I	1.3	0.3	3.4	2.4	34.7
EHEC H7 Wt	Curly II	0.8	0.2	4.2	3.8	42.1
Achromobacter	Normal	2.4	0.4	-2.0	1.1	29.5
Achromobacter	Normal II	1.7	0.4	2.3	1.8	38.9
Achromobacter	Semi-coiled	1.3	0.5	1.8	2.2	52.1
E. coli K12	Normal	2.5	0.4	2.0	1.0	27.2
E. coli K12	Semi-coiled	1.3	0.6	1.7	2.2	53.5
E. coli K12	Curly I	0.9	0.3	3.0	3.4	49.2
E. coli K12	Curly II	0.8	0.2	4.5	3.9	40.8

Supplementary Table 3. Structural homologs to flagellin outer domains in this study.

Outer domain	Homolog 1 *	Homolog 1 PDB ID	Homolog 1 Z-score	Homolog 2 *	Homolog 2 PDB ID	Homolog 2 Z-score
S. meliloti D2	RRNA 2'-O-METHYLTRANSFERASE FIBRILLARIN	6zdt	3	2-5A-DEPENDENT RIBONUCLEASE	4oau	2.5
EHEC H7 D2	FRPC OPERON PROTEIN	5edf	3.8	CYTOTOXIC TRANSLATIONAL REPRESSOR OF TOXIN-ANTITO	5mje	3.6
EHEC H7 D3	HYPOTHETICAL PROTEIN YIIX	2if6	3.9	S-LAYER PROTEIN	3cvz	3.9
EHEC H7 D4	H1 RNA	6ahr	4.3	TYPE II SECRETION SYSTEM PROTEIN E, HEMOLYSIN-COR	4ksr	3.7
Achromobacter D2	FRPC OPERON PROTEIN	5edf	3	ADENYLATE CYCLASE EXOY	5xnw	2.9
Achromobacter D3	DE NOVO DESIGNED PROTEIN FOLDIT3	6msp	4.4	MACHADO-JOSEPH DISEASE PROTEIN 1	2aga	4.4
Achromobacter D4	STAGE II SPORULATION PROTEIN SA	3o6q	3.9	UNCHARACTERIZED PROTEIN	3fb9	3.5
EPEC H6 D2	PUTATIVE ENZYME RELATED TO ALDOSE 1-EPIMERASE	2hta	2.4	DNA PRIMASE/HELICASE	6n9x	2.1
EPEC H6 D3	BSHC	4wbd	3.5	GLUTATHIONE SYNTHETASE	1gsa	3.1
EPEC H6 D4	CDIA TOXIN	5t86	4.4	REPLICATION PROTEIN B	2h20	3.7

* The first and second highest hits are shown for each domain.

Supplementary Table 4. Comparison of the interfacial areas of adjacent flagellin subunits from flagellar filament structures.

**Bacterial species**	**S_n:S_{n+5} interfacial surface area (Å^2)**	**S_n:S_{n+11} interfacial surface area (Å^2)**
S. meliloti	2166.6	2086.8
EHEC H7	2061.8	1910.1
Achromobacter sp. MFA1 R4	2374.5	2074.3
Campylobacter jejuni	2137.1	2816.4
Salmonella typhimurium FljB (R-type)	1967.9	1780.4
Bacilis subtilis (L-type)	1822.7	1876.5

Supplementary Table 5. Primers used in this study.

EHEC H7 FliC deletion primer forward	ATCATGGCACAAGTCATTAATACCAACAGCCTCTCGCTGATCACTCAAAAGTGTAGGCTGGAGCTGCTTC
EHEC H7 FliC deletion primer reverse	TGGCGTTGCCGTCAGTCTCAGTTAATCAGGTTACAACGATTAACCCTGCACATATGAATATCCTCCTTAG
H7 fliC complement forward	ACGGTACCAAATGGCTGTTTGTGAAA
H7 fliC complement reverse	ACAAGCTTAAATTGCGCTTTATGTTG
H7 mutation NNFF forward	GACTTTCAACGCTGGTAGCGCAGCT
H7 mutation NNFF reverse	GTCAAGAAGCCTGCATCGTCTACGTATG
H7 mutation NNRR forward	GACTCGCAACGCTGGTAGCGCAGCT
H7 mutation NNRR reverse	GTCAAGCGGCCTGCATCGTCTACGTATG
FliC complementation into E-coli K-12 forward	GGTGGTGAATTCTGAAATACTTGCCATGCG-EcoRI
FliC complementation into E-coli K-12 reverse	GGTGGTGTCGACTTAACCCTGCAGCAGAGA-SalI

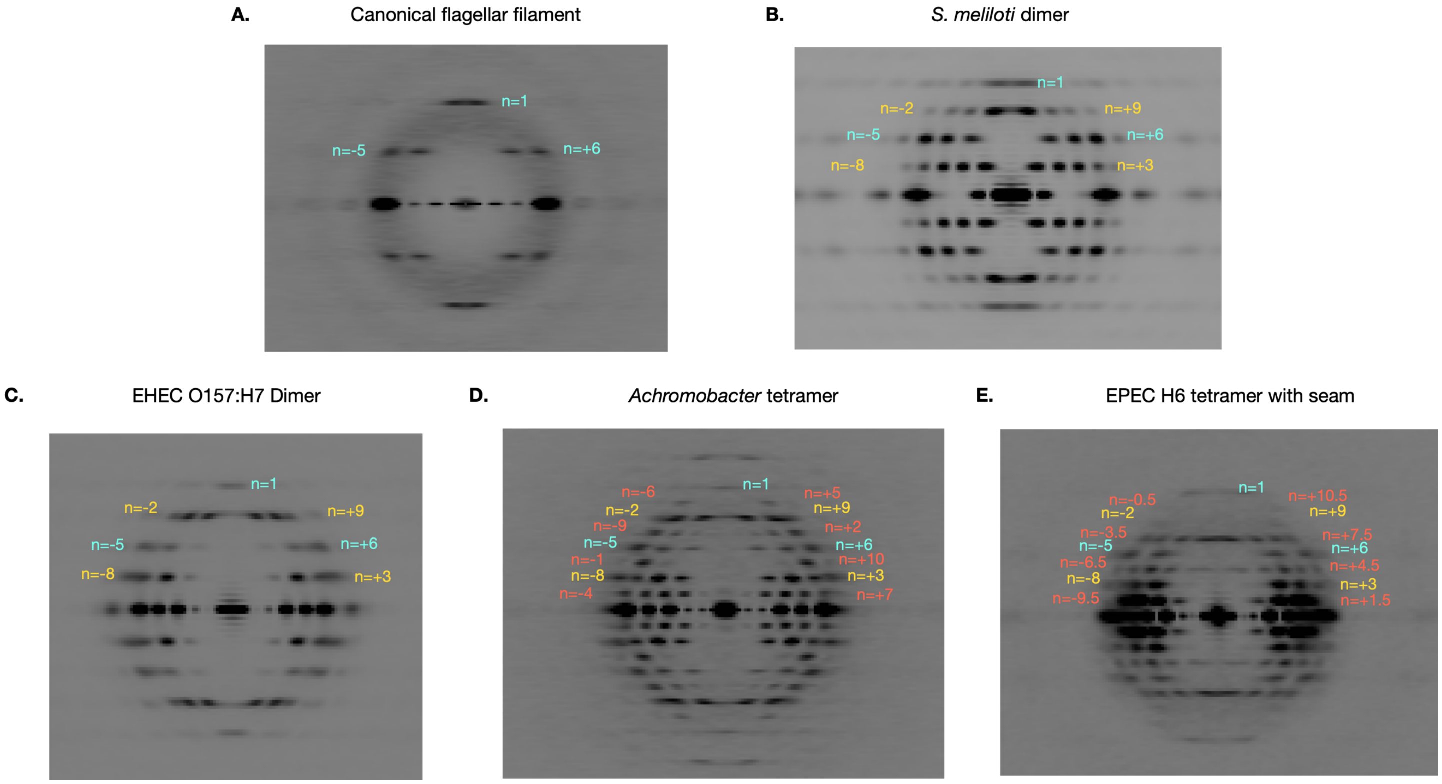

Supplementary Fig. 1. Power spectra of canonical (monomeric), dimeric, and tetrameric flagellar filaments. A. Averaged power spectrum of canonical flagellar filament form *Agrobacterium tumefaciens*. The power spectrum matches those published for wild-type supercoiled flagellar filaments[13]. The n=-5 and n=+6 layer lines are not separated due to the fact that the average twist is close to 65.45°, which means that the 11-start protofilaments are nearly vertical. **B.** Averaged power spectrum of the *S. meliloti* dimeric flagellar filament. There is a doubling of the number of observed layer lines compared to the canonical filament due to a reduction in symmetry along the 6-start helix, giving rise to the 3-start helices. **C.** Averaged power spectrum of the dimeric EHEC O157:H7 flagellar filament. **D.** Averaged power spectrum of the tetrameric *Achromobacter* flagellar filament. This *Achromobacter* filament has four times the number of observed layer lines compared to the canonical flagellar filament. A tetramer is created by a reduction of symmetry along the 8-start (n=8) helix. **E.** Averaged power spectrum of the EPEC O127:H6 flagellar filament. This power spectrum also has four times the number of observed layer lines as the canonical flagella. However, its pattern is distinct from the *Achromobacter* due to a reduction in symmetry along the 9-start helix rather than the 8-start. This generates layer lines with half-integer Bessel orders

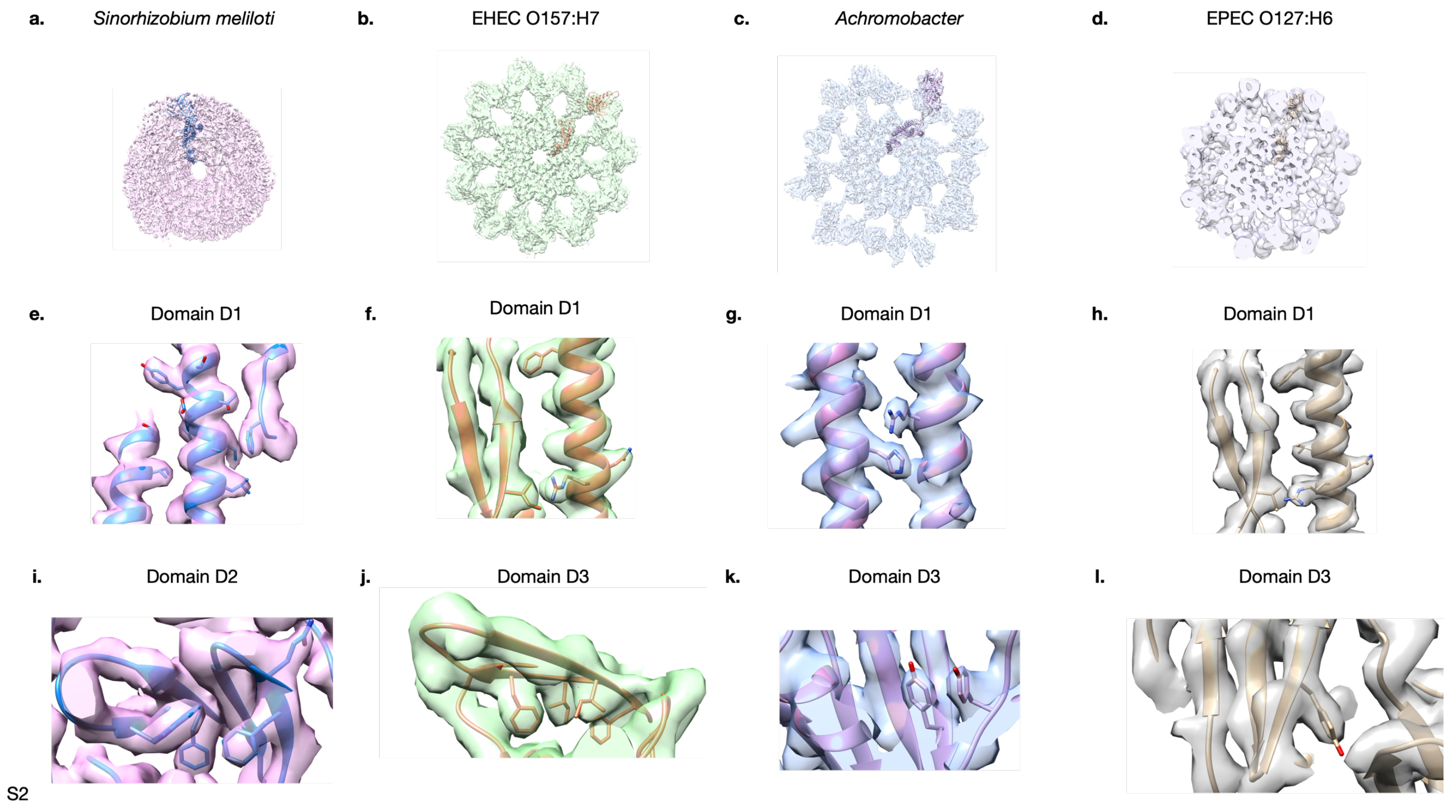

Supplementary Fig. 2. CryoEM density maps of screw-like and ODS flagellar filaments.

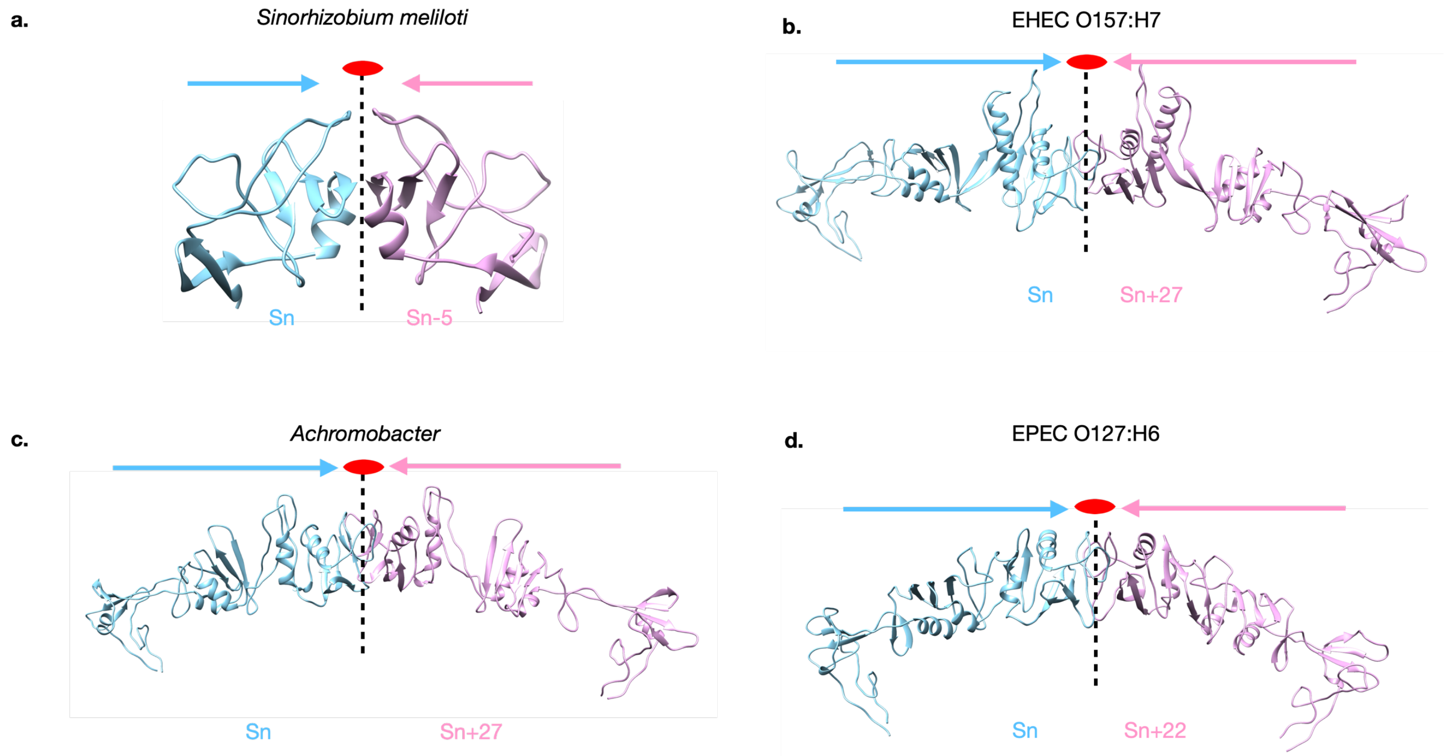

Supplementary Fig. 3. Outer domain dimers of EHEC O157:H7, EPEC O127:H6, *Achromobacter*, and *S. meliloti* flagellar filaments. Arrows pointing towards each other indicate that the outer domains have D1 symmetry and are identical to each other through a 180° rotation about the vertical 2-fold axis shown. Since these 2-fold axes intersect the helical axis of the filament, the outer surfaces of the flagellar filaments have a global D1 symmetry.

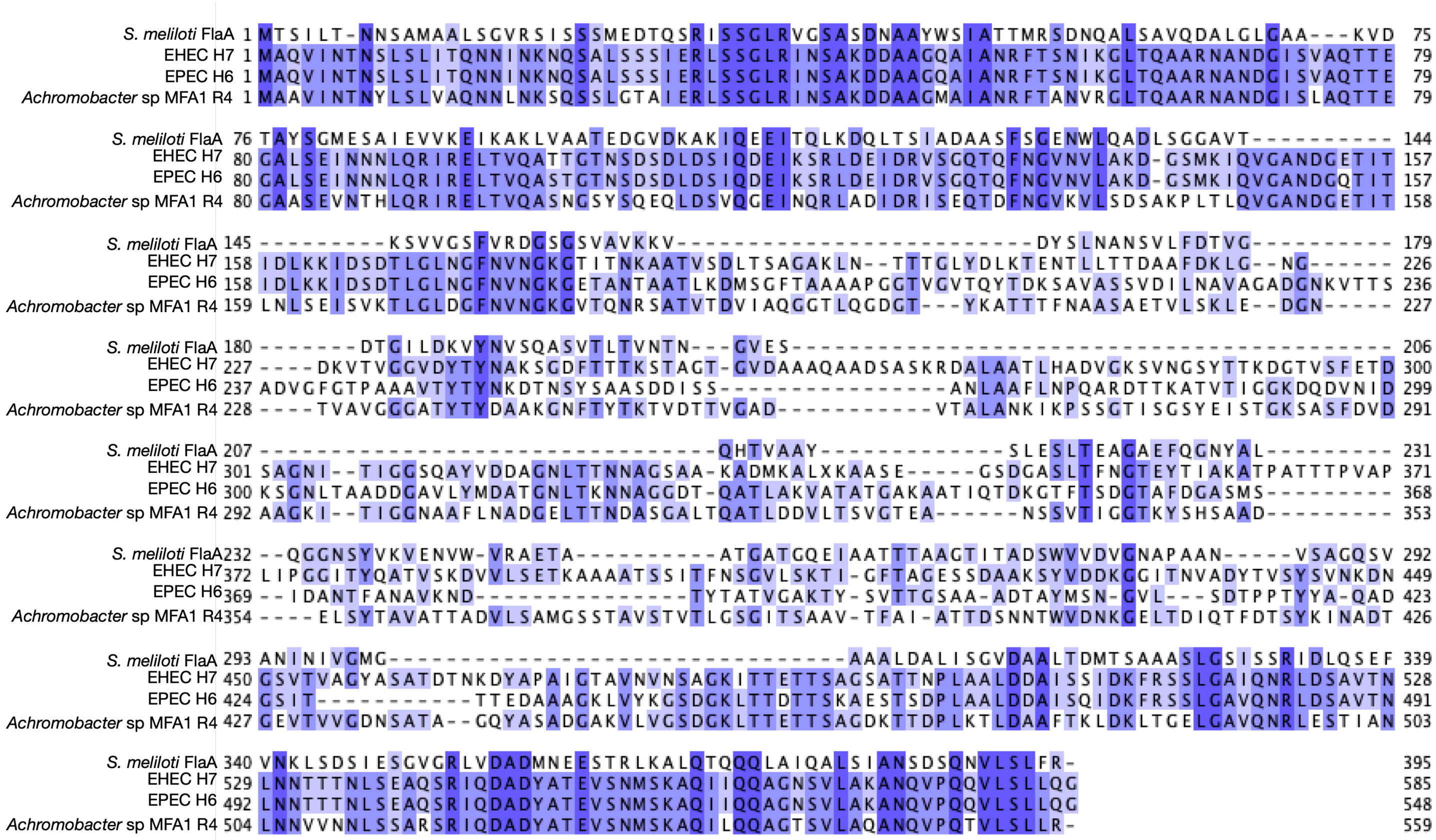

Supplementary Fig. 4. Multiple sequence alignment of the flagellin subunits from the structures in this study. The alignment is colored according to percentage identity.

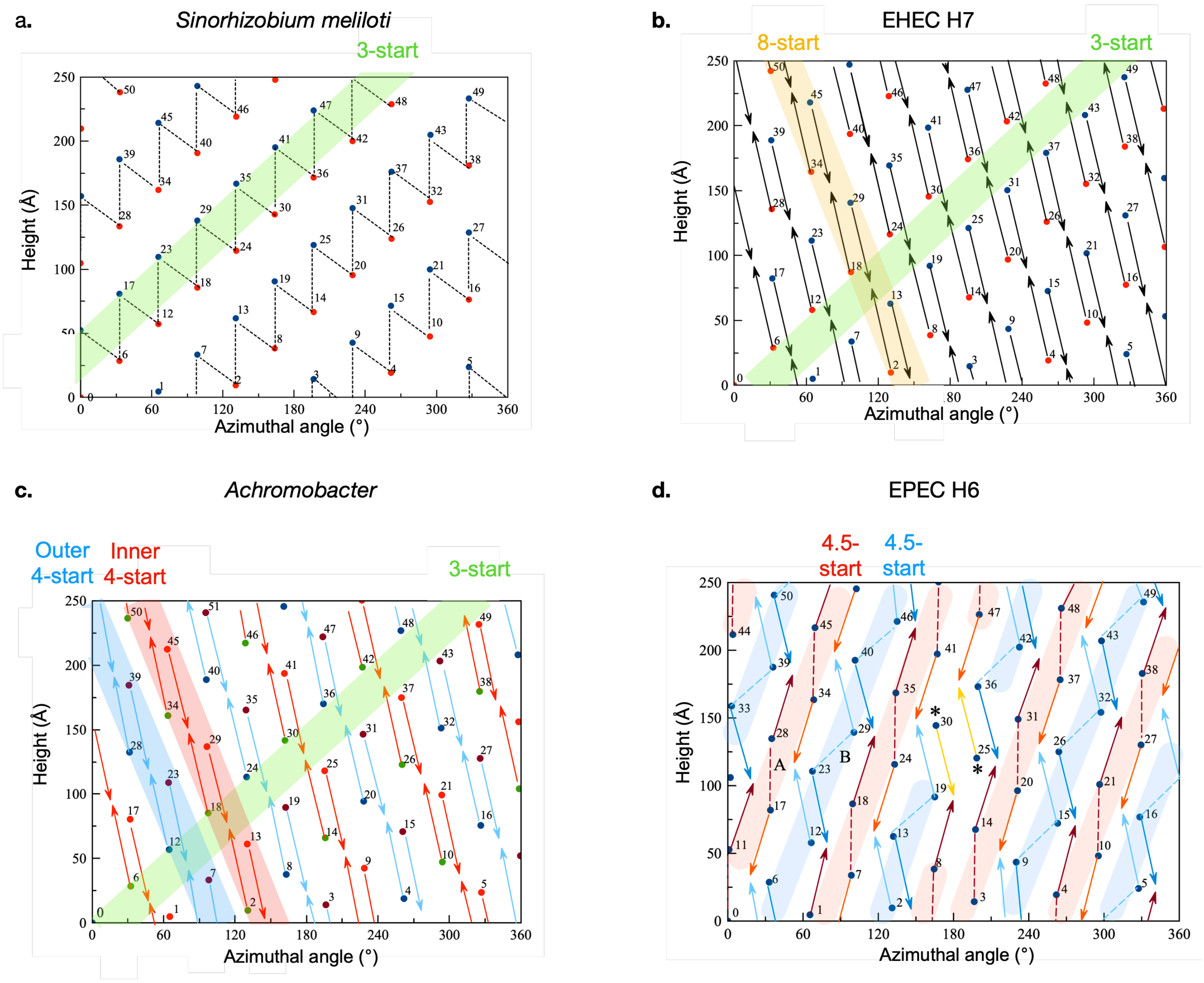

Supplementary Fig. 5. Helical nets of the screw-like and outer domain sheathed flagellar filaments. a. Helical net of the *S. meliloti* flagellar filament. We use the convention throughout that the surface has been unrolled and one is looking at it from the outside. Alternating blue and red dots indicate the two different flagellin conformations (up or down). The S_n: S_{n+5} and S_n: S_{n+11} dimers are indicated by dashed lines and generate the 3-start helix (green highlight), which is a pairing across the 6-start helix of the core symmetry. **b.** Helical net of the EHEC H7 flagellar filament. Arrows indicate “up” or “down” conformation and meet at the location of each S_n: S_{n+27} dimer. The 3-start helix formed by this dimer is shown in green. The 8-start is shown in yellow. **c.** Helical net of the *Achromobacter* flagellar filament. Red and blue arrows and highlights show the two different radii for the S_n: S_{n+27} dimers creating the two different 4-start helices. The right-handed 3-start helix is shown in green. **d.** Helical net of the EPEC H6 flagellar filament. Dashed lines indicate domain D2 dimers and arrows indicate domain D4 dimers. The blue and red helices are broken at the seam, *, indicated by the yellow arrows and the absence of dashed lines.

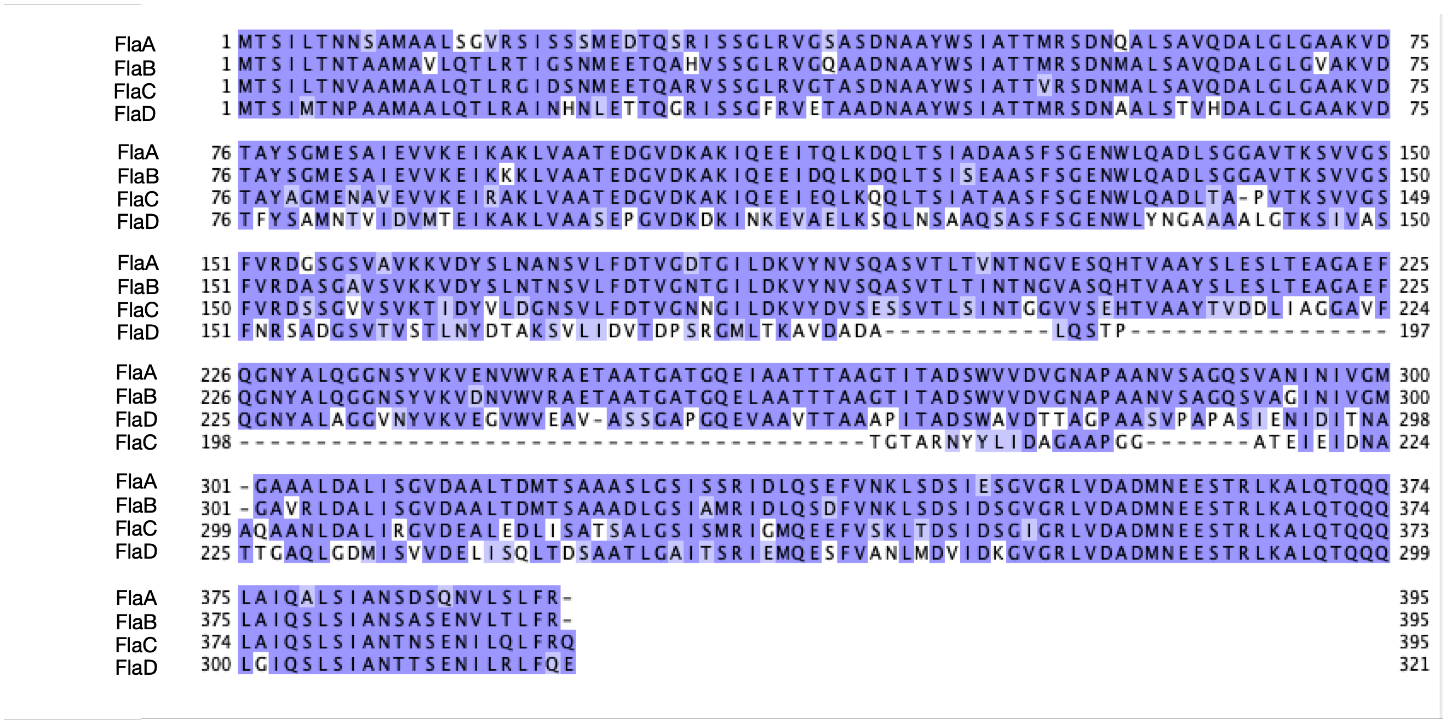

Supplementary Fig. 6 Sequence alignment of the *S. meliloti* flagellins. Alignment is colored by percent identity.

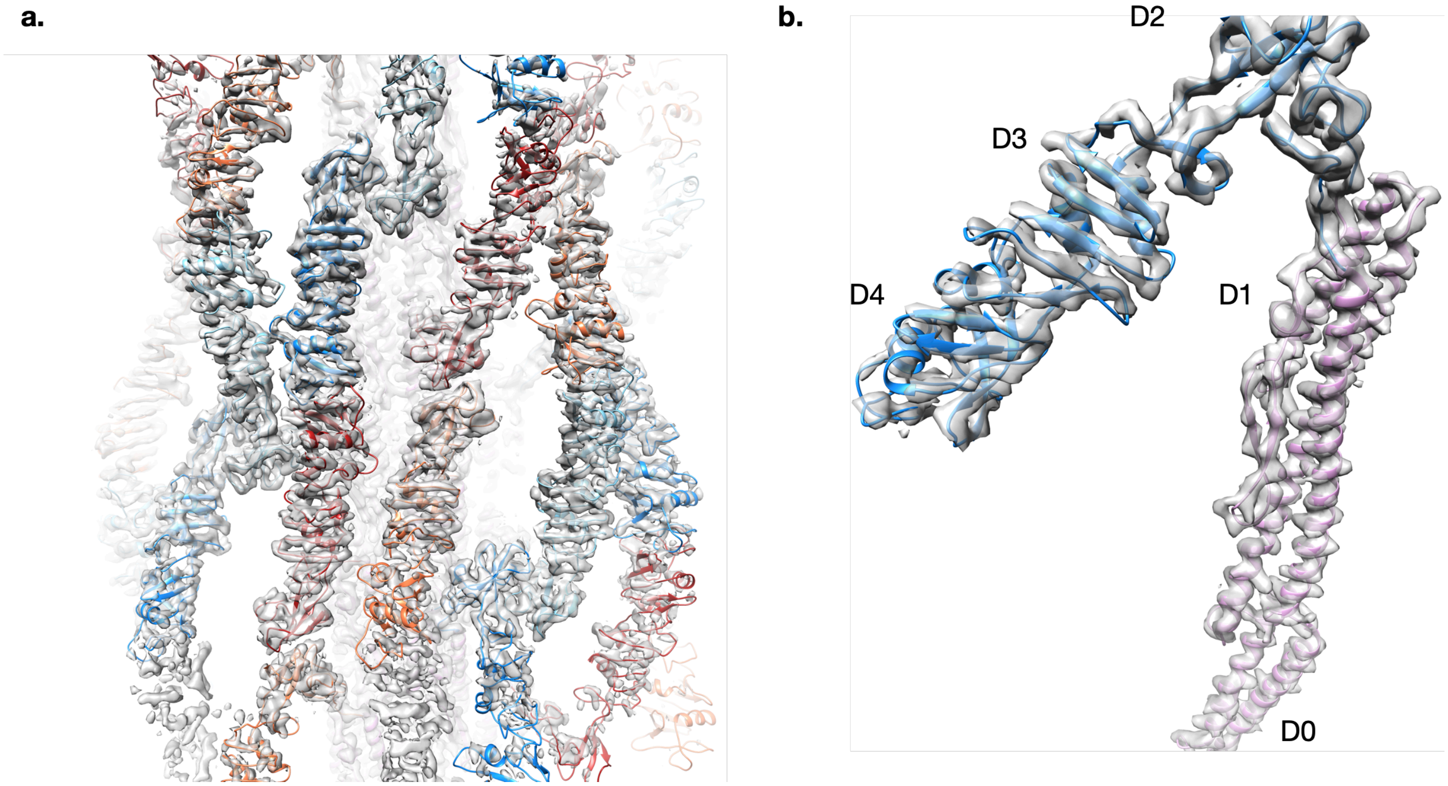

Supplementary Fig. 7 High resolution structure of the EPEC O127:H6 sheathed flagellar filament. a. Surface of the EPEC H6 flagellar filament density map with fit of atomic models. **b.** Full atomic model and corresponding density map for a single EPEC H6 subunit.

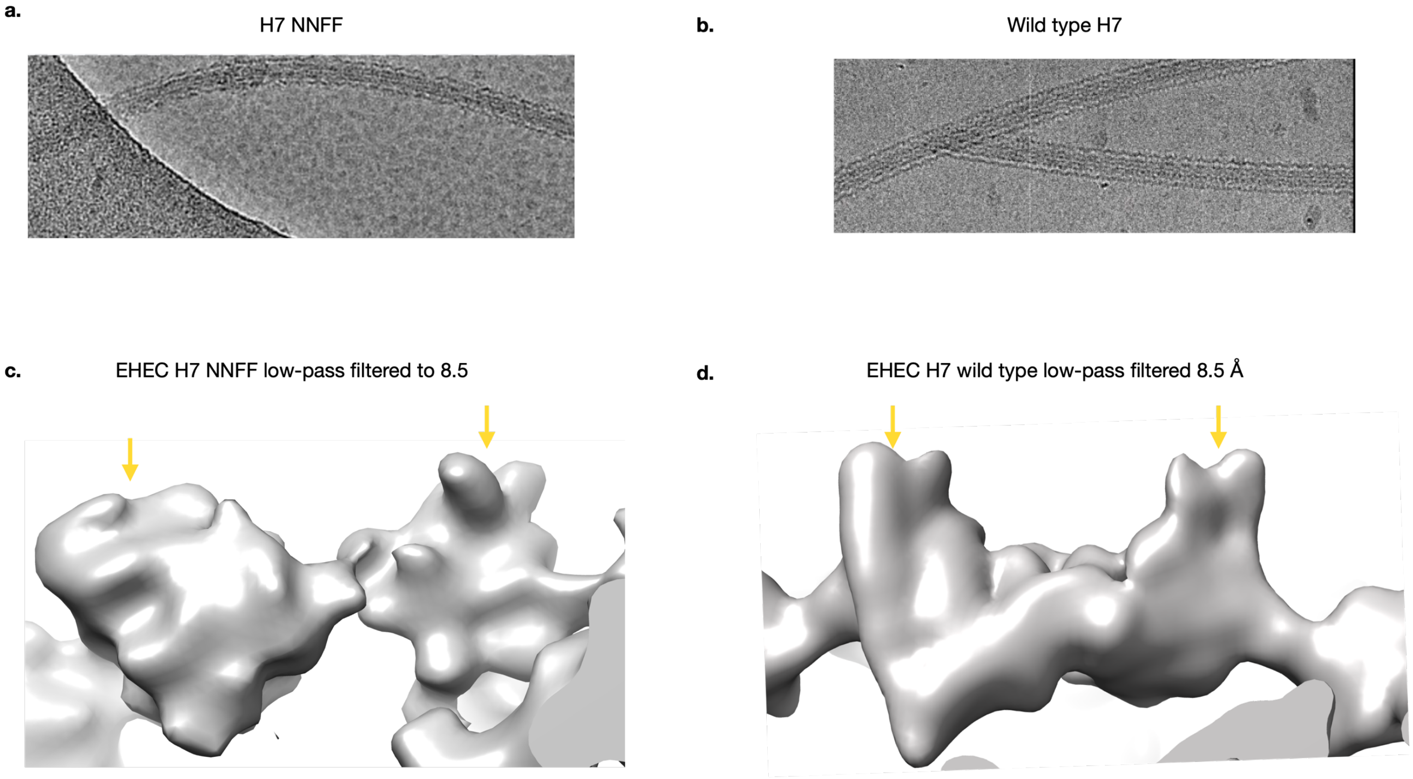

S6

Supplementary Fig. 8. Structural comparison of the mutant FF H7 flagellar filaments with wild type. a. Cryo-electron micrograph of mutant FF flagellar filaments. **b.** Cryo-electron micrograph of wild type H7 flagellar filaments. **c.** Low-pass filtered (8.5 Å) structure of the FF mutant flagellar filament. Arrows point to ~30 Å spacing between the largest radii region of the two dimer subunits. **d.** The EHEC H7 wild-type density map low-pass filtered to 8.5 Å resolution for comparison. The yellow arrows point to the same 30 Å spacing as in C.

a. Wild type H7

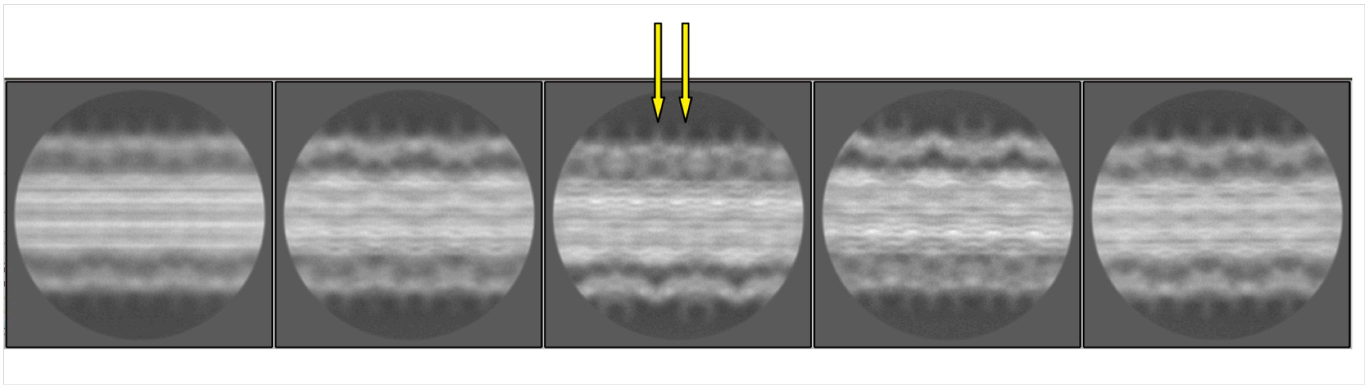

b. H7 NNFF

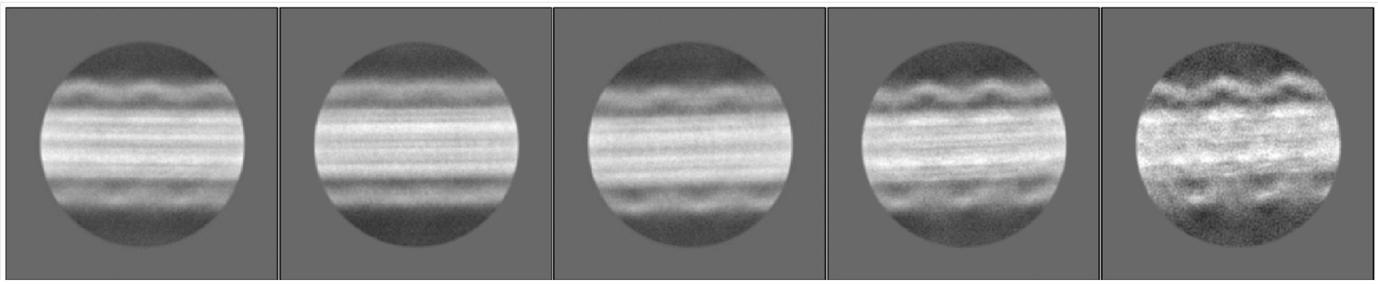

Supplementary Fig. 9. 2D class averages of wild type EHEC H7 and mutant FF H7 flagellar filament. a. Class averages of the wildtype H7 flagellar filament. The ~30 Å spacing shown in Fig 7D is apparent in the 2D averages and is shown with the yellow arrows. The wildtype H7 class averages were obtained from 20,000 particles. **b.** Class averages of the EHEC H7 FF mutant flagellar filament. There are no defined 30 Å features corresponding to the spacing in the wild type. The FF mutant class averages were obtained from ~18,000 particles.

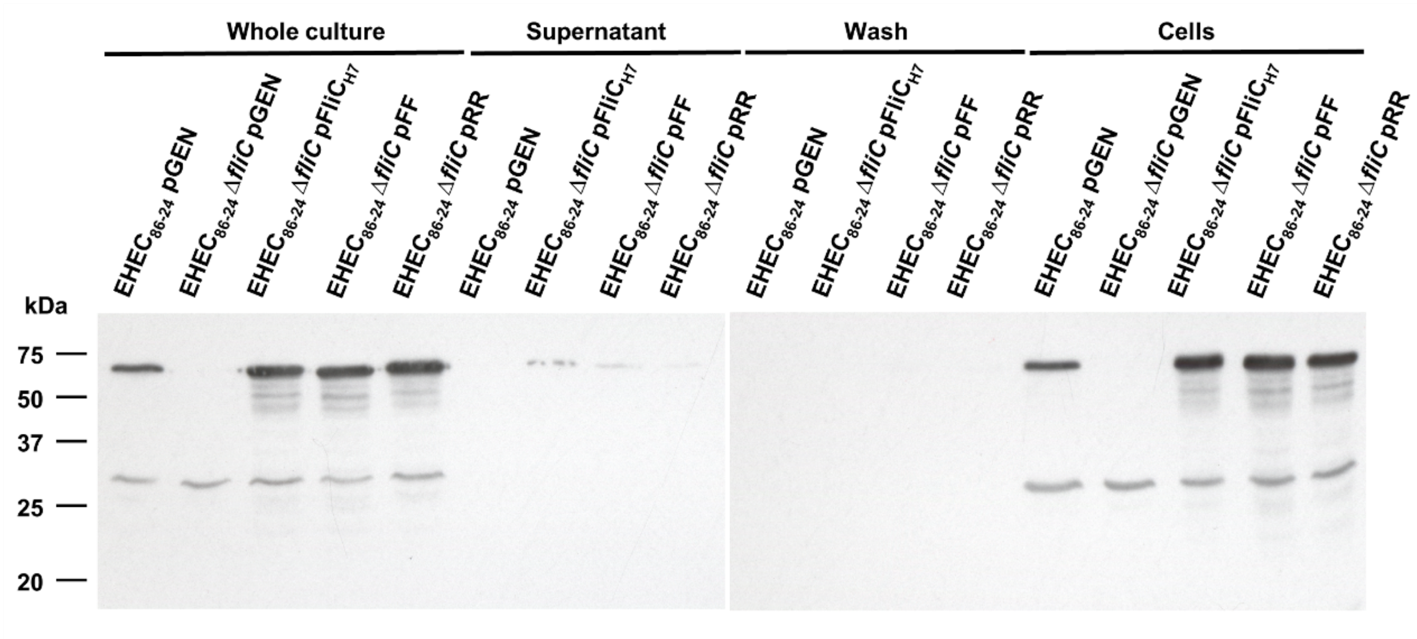

Supplementary Fig. 10. The FliC$_{NN\rightarrow FF}$ and FliC$_{NN\rightarrow RR}$ mutations do not affect flagellum production or filament stability. FliC western blot analysis of cellular flagellin levels in EHEC 86-24 mutants producing FliC$_{NN\rightarrow FF}$ (pFF) and FliC$_{NN\rightarrow RR}$ (pRR). Motile bacteria were cultured and washed as described in Materials and Methods and samples of the whole culture, culture supernatant, pellet wash, and suspended cells were mixed with equal volumes of Laemmli buffer, boiled for 10 minutes, and detected via western blot analysis with an α-flagellin$_{K\text{-}12}$ antiserum. FliC$_{H7}$ is detected at a molecular weight of approximately 60 kDa. The non-specific cross-reaction band at approximately 30 kDa served as a loading control.

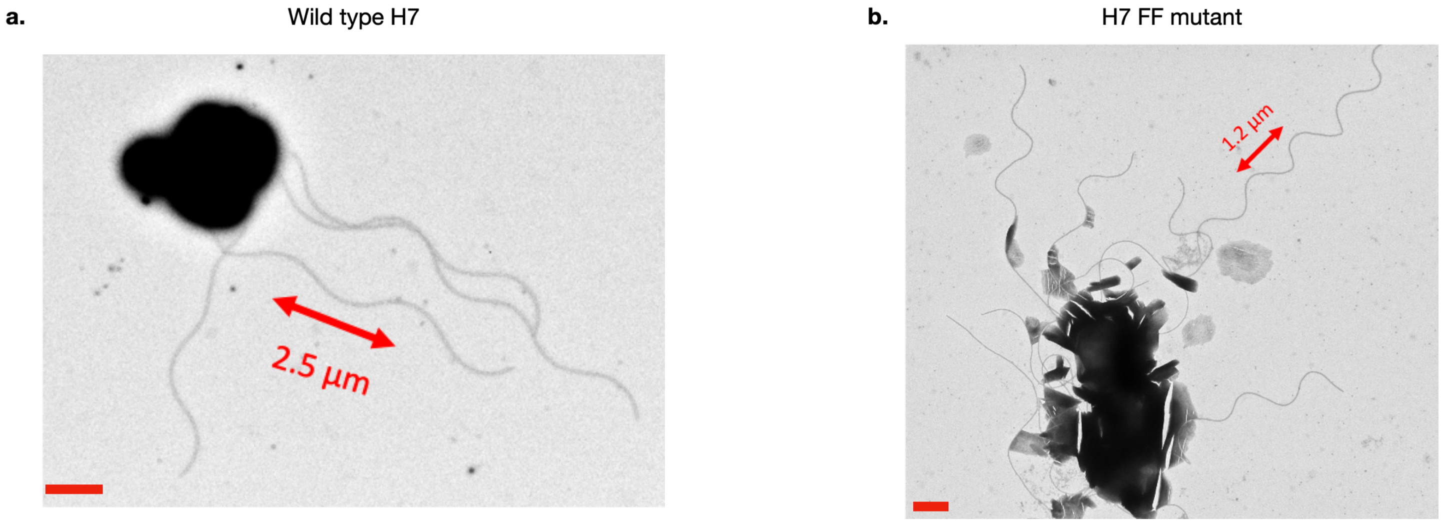

Supplementary Fig. 11. Negative stain TEM of cells with wildtype H7 and mutant H7 FF flagellar filaments. a. Micrograph showing wildtype EHEC cells with their flagellar filaments. The red double arrows indicate a distance of 2.5 µm. The scale bar in the bottom left of the image corresponds to ~1 µm. **b.** Micrograph showing mutant FF EHEC cells and their flagellar filaments. The scale bar in the bottom

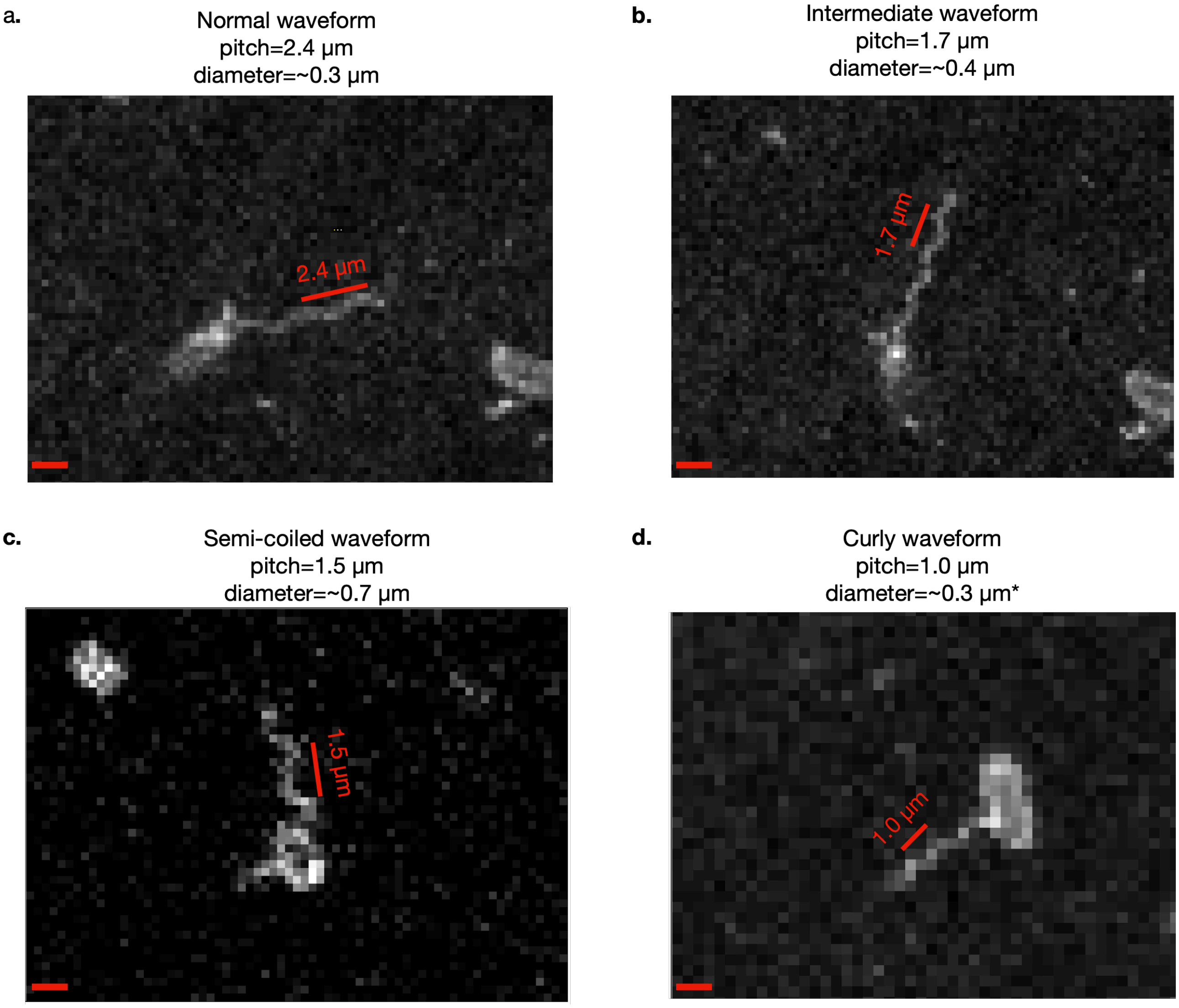

Supplementary Fig. 12 Observed waveforms of labeled EHEC O157:H7 flagellar filaments. a-d. Fluorescence microscopy images of wildtype EHEC O157:H7 cells ($EHEC_{86\text{-}24}$) with themselves and flagella labeled with Alexafluor 546 dye. The red scale bar in the bottom left of each image corresponds to ~1 µm. **a.** An EHEC O157:H7 cell with its flagellar filament adopting the normal waveform. **b.** An EHEC O157:H7 cell with its flagella in the intermediate waveform. **c.** An EHEC O157:H7 cell with its flagellar adopting a semicoiled waveform. **d.** An EHEC O157:H7 cell with its flagellar filament in the curly waveform.

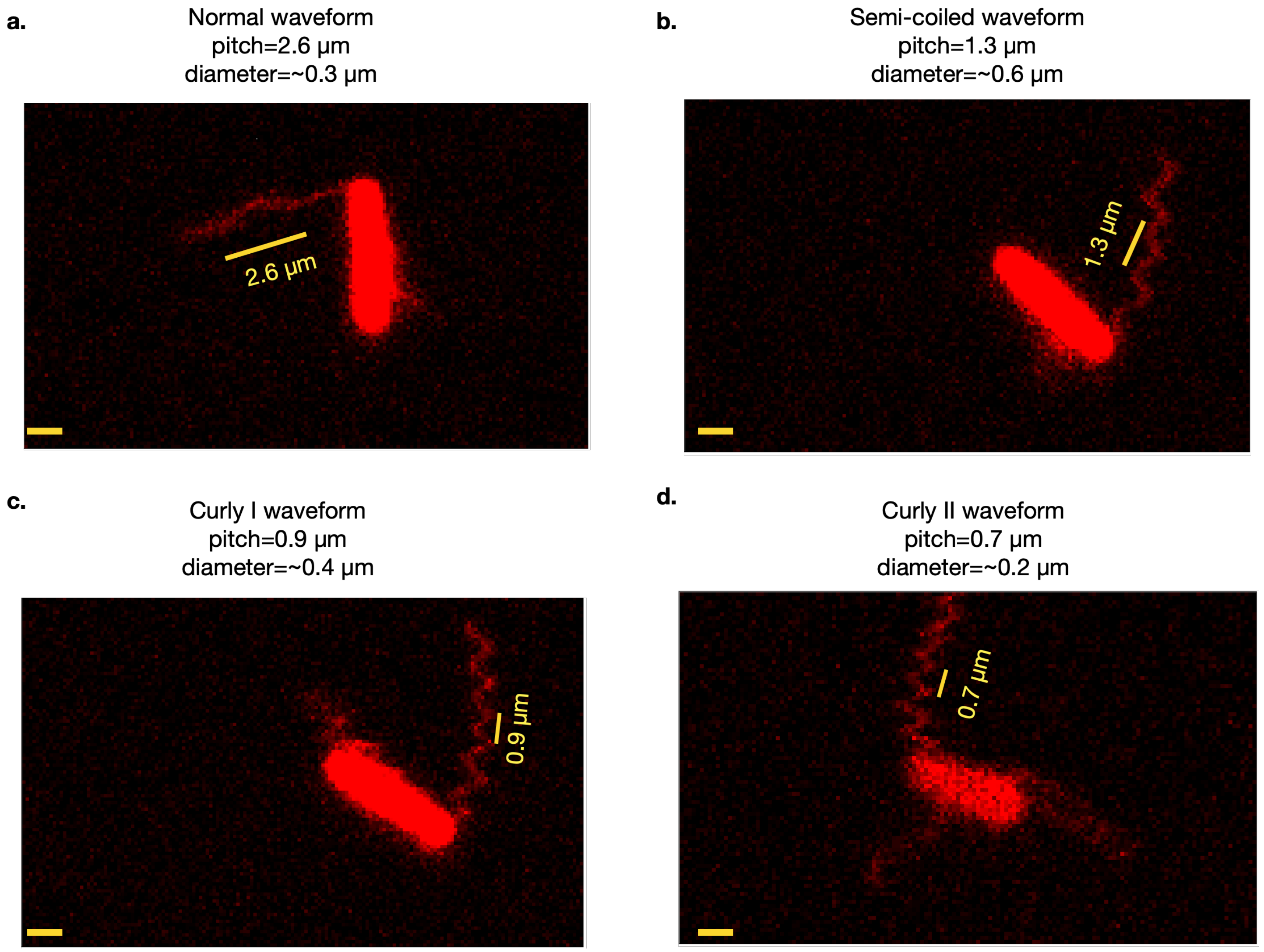

Supplementary Fig. 13. Observed waveforms of *E. coli* K-12 AW405 flagellar filaments. a-d. Fluoresecnce microscopy images of wildtype *E. coli* K-12 AW405 cells and their flagellar filaments labeled with AlexaFluor 547 dye. The yellow scalebar in the bottom left of each image is ~1 μm in length. **a.** Example of the normal waveform of the *E. coli* K-12 flagellar filament. **b.** Example of the semi-coiled waveform of the *E. coli* K-12 flagellar filament. **c.** Example of the curly I waveform of the *E. coli* K-12 flagellar filament. **d.** Example of the curly II waveform of the *E. coli* K-12 flagellar filament.

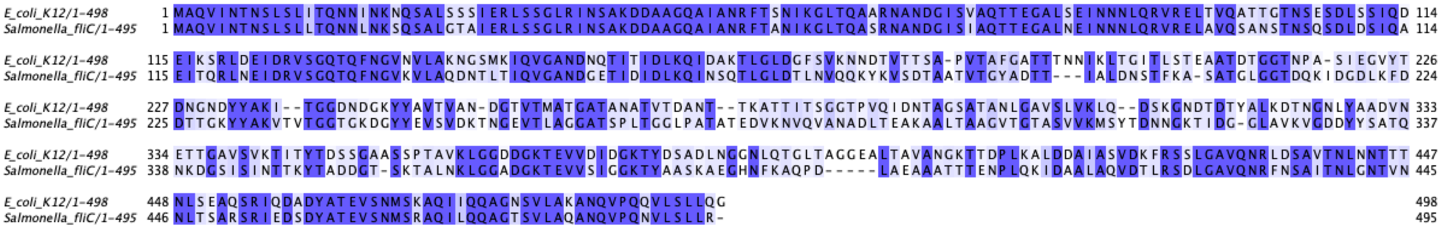

Supplementary Fig. 14. Sequence alignment of the *E. coli* K-12 AW405 flagellin and *S. typhimurium* LT2 phase 1 flagellin. Alignment is colored by degree of physicochemical conservation of side chain residues, with identical residues shaded dark blue.

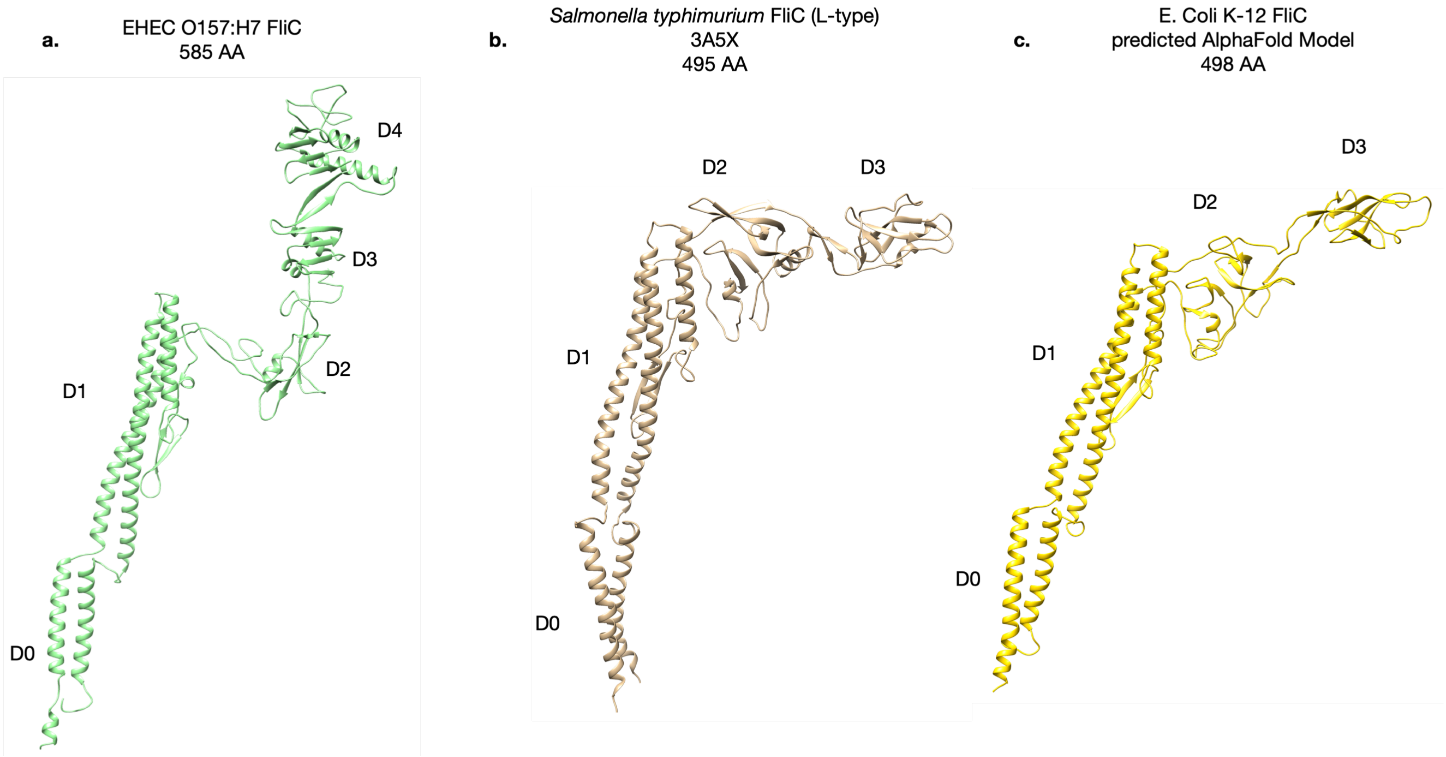

Supplementary Fig. 15. Comparison of EHEC H7, *S. typhimurium*, and AlphaFold-predicted *E. coli* K-12 flagellin models. a. Atomic model of the EHEC H7 flagellin determined in this study. **b.** Atomic model of the *S. typhimurium* flagellin[14]. **c.** Predicted atomic model for the *E. coli K-12* flagellin created with AlphaFold[50].

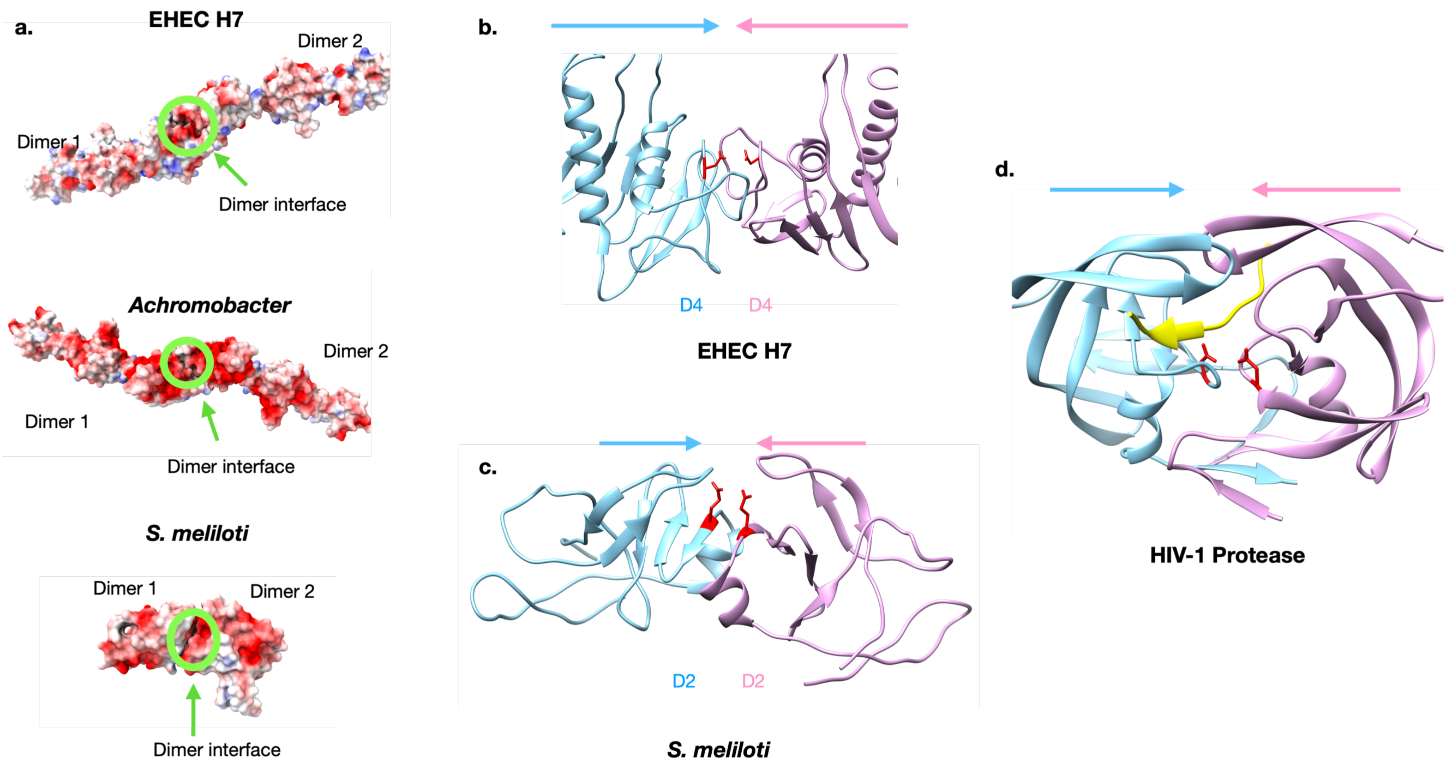

Supplementary Fig 16. Negatively charged interfaces of the outer domain dimers. a. Electrostatic potential surfaces of the EHEC H7, *Achromobacter,* and *S. meliloti* dimers. Red indicates negatively charged surfaces, white indicates neutral charged surfaces, and blue indicates positively charged surfaces. **b.** Interfacing aspartate 316 residues of the EHEC H7 domain D4 dimer. The aspartate 316 residues are colored red. **c.** Interfacing glutamate 224 residues (red) of the *S. meliloti* domain D2 dimer. **d.** The HIV-1 aspartic protease (1DMP). The catalytic residues are colored red.

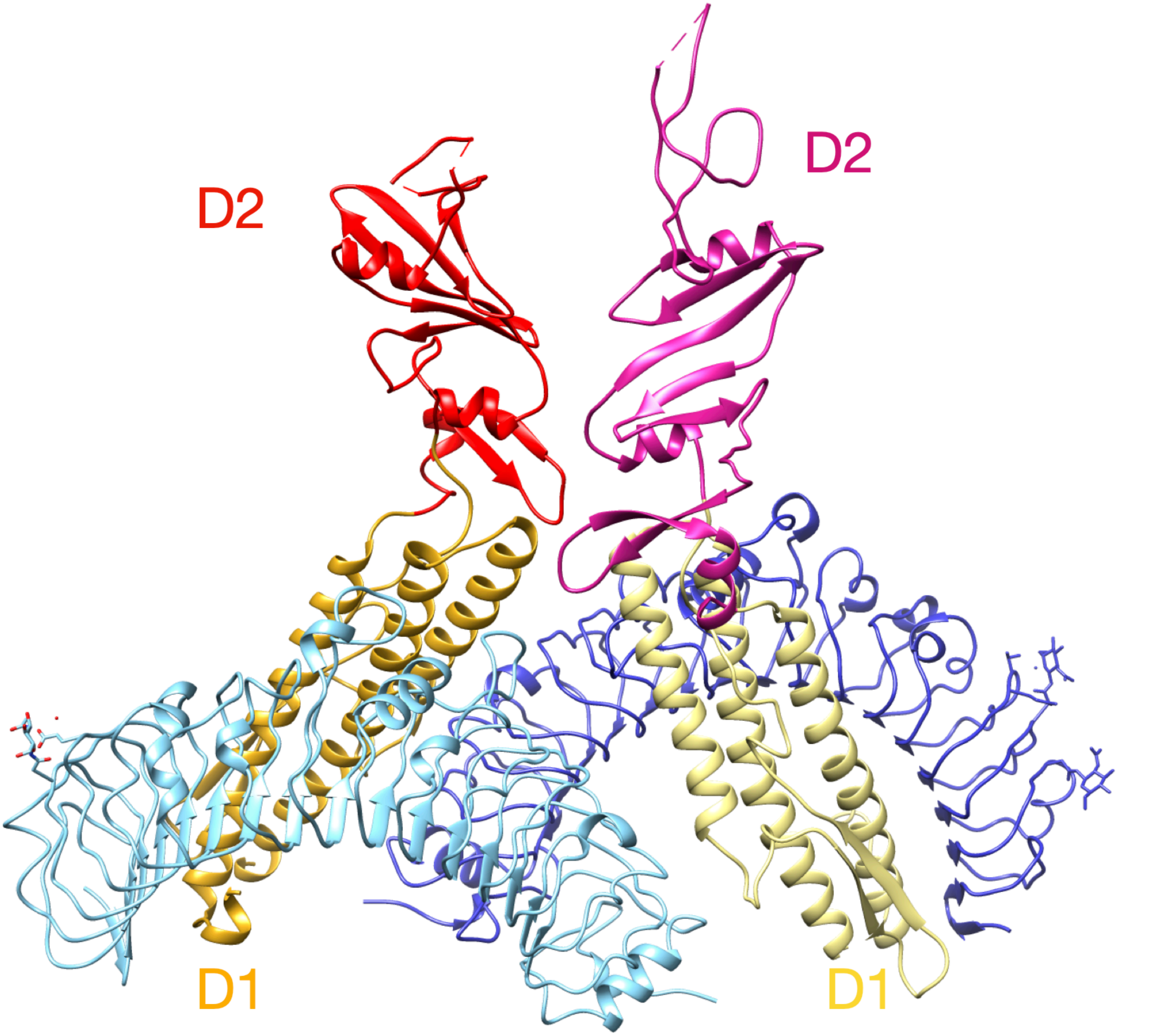

Supplementary Fig 17. Binding of TLR5 to flagellin monomers. Crystal structure (3V47) showing a complex consisting of two TLR5:flagellin heterodimers that bind two each other[70]. The TLR5 ectodomains are colored blue. The *S. typhimurium* flagellins are colored gold or tan in domain D1 and red or violet in domain D2.
