# Supplementary material for: Flagellin outer domain dimerization modulates motility in pathogenic and soil bacteria from viscous environments
Source: Nat Commun. 2022 Mar 17;13:1422. doi: 10.1038/s41467-022-29069-y (PMC8931119; doi:10.1038/s41467-022-29069-y)
Supplement: Supplementary file 2 — Description of Additional Supplementary Files [file 41467_2022_29069_MOESM2_ESM.pdf]

Description of Additional Supplementary Files

File name: Supplementary Movie 1

Description: Fluorescence microscopy of EHEC O157:H7 cell with two labeled flagellar filaments. Each frame is 210 milliseconds. The various discernable waveforms are indicated in each frame. N is normal; I is intermediate; SC is semi-coiled; C is curly.

File name: Supplementary Movie 2

Description: Fluorescence microscopy of EHEC O157:H7 cell with many labeled flagellar filaments. Each frame is 210 milliseconds. The cell undergoes two tumbles. Tumble 1 (T1) is about 420 ms long. Tumble 2 (T2) is about 630 ms long.

File name: Supplementary Movie 3

Description: Fluorescence microscopy video of EHEC FF mutant cell with labeled flagella. Each frame is 210 milliseconds.

File name: Supplementary Movie 4

Description: Fluorescence microscopy of labeled rotating *Achromobacter* sp. MFA1 R4 flagellar filaments. Each frame is 240 milliseconds.

File name: Supplementary Movie 5

Description: Fluorescence microscopy of labeled rotating wild-type EHEC H7 flagellar filaments. Each frame is 210 milliseconds.

File name: Supplementary Movie 6

Description: Fluorescence microscopy of labeled rotating EHEC FF flagellar filaments. Each frame is 210 milliseconds.

File name: Supplementary Movie 7

Description: Phase contrast microscopy of wild-type *E. coli* K-12 AW405 cells. Each frame is 33 milliseconds.

File name: Supplementary Movie 8

Description: Phase contrast microscopy of wild-type EHEC O157:H7 cells. Each frame is 33 milliseconds.

File name: Supplementary Movie 9

Description: Phase contrast microscopy of EHEC FF mutant cells. Each frame is 33 milliseconds long. The arrow which movies at various time points is always pointing at the same bacterium.
