# Supplementary material for: Flagellin outer domain dimerization modulates motility in pathogenic and soil bacteria from viscous environments
Source: Nat Commun. 2022 Mar 17;13:1422. doi: 10.1038/s41467-022-29069-y (PMC8931119; doi:10.1038/s41467-022-29069-y)
Supplement: Supplementary file 12 — Reporting Summary [file 41467_2022_29069_MOESM12_ESM.pdf]

Corresponding author(s): Egelman, Edward H.

Last updated by author(s): 3 February 2022

Reporting Summary

Nature Portfolio wishes to improve the reproducibility of the work that we publish. This form provides structure for consistency and transparency in reporting. For further information on Nature Portfolio policies, see our Editorial Policies and the Editorial Policy Checklist.

Statistics

For all statistical analyses, confirm that the following items are present in the figure legend, table legend, main text, or Methods section.

n/a	Confirmed	
☐	☒	The exact sample size (*n*) for each experimental group/condition, given as a discrete number and unit of measurement
☐	☒	A statement on whether measurements were taken from distinct samples or whether the same sample was measured repeatedly
☐	☒	The statistical test(s) used AND whether they are one- or two-sided<br>*Only common tests should be described solely by name; describe more complex techniques in the Methods section.*
☒	☐	A description of all covariates tested
☒	☐	A description of any assumptions or corrections, such as tests of normality and adjustment for multiple comparisons
☐	☒	A full description of the statistical parameters including central tendency (e.g. means) or other basic estimates (e.g. regression coefficient) AND variation (e.g. standard deviation) or associated estimates of uncertainty (e.g. confidence intervals)
☐	☒	For null hypothesis testing, the test statistic (e.g. *F*, *t*, *r*) with confidence intervals, effect sizes, degrees of freedom and *P* value noted<br>*Give P values as exact values whenever suitable.*
☒	☐	For Bayesian analysis, information on the choice of priors and Markov chain Monte Carlo settings
☒	☐	For hierarchical and complex designs, identification of the appropriate level for tests and full reporting of outcomes
☒	☐	Estimates of effect sizes (e.g. Cohen's *d*, Pearson's *r*), indicating how they were calculated

Our web collection on statistics for biologists contains articles on many of the points above.

Software and code

Policy information about availability of computer code

Data collection	EPU (ThermoFisher Scientific), CryoSPARC v3.3
Data analysis	CryoSPARC v3.3, Relion v3.1, Coot v0.9, Rosetta CM, Phenix, Spider v22.10

For manuscripts utilizing custom algorithms or software that are central to the research but not yet described in published literature, software must be made available to editors and reviewers. We strongly encourage code deposition in a community repository (e.g. GitHub). See the Nature Portfolio guidelines for submitting code & software for further information.

Data

Policy information about availability of data

All manuscripts must include a data availability statement. This statement should provide the following information, where applicable:

- Accession codes, unique identifiers, or web links for publicly available datasets
- A description of any restrictions on data availability
- For clinical datasets or third party data, please ensure that the statement adheres to our policy

All atomic models were deposited in the Protein Data Bank and all density maps were deposited in the Electron Microscopy Database. The S. meliloti map and model are available as EMD-25215 [https://www.ebi.ac.uk/pdbe/entry/emdb/EMD-25215] and PDB 7SN9 [http://doi.org/10.2210/pdb7SN9/pdb]. The EHEC H7 full filament map and model are available as EMD-25211 [https://www.ebi.ac.uk/pdbe/entry/emdb/EMD-25211] and PDB 7SN4 [http://doi.org/10.2210/pdb7SN4/pdb]. The EHEC H7 outer domain sheath with D1 symmetry applied is available as EMD-25388 [https://www.ebi.ac.uk/pdbe/entry/emdb/EMD-25388]. The EHEC H7 FF mutant structure is available as EMD-25212 [https://www.ebi.ac.uk/pdbe/entry/emdb/EMD-25212]. The EPEC H6 high resolution map and model are available as EMD-25213 [https://www.ebi.ac.uk/pdbe/entry/emdb/EMD-25213] and PDB 7SN7 [http://doi.org/10.2210/pdb7SN7/pdb]. The lower resolution EPEC H6 map is available as EMD-25386 [https://www.ebi.ac.uk/pdbe/entry/emdb/EMD-25386], while the model of the two seam subunits is available as PDB 7SQJ [http://

doi.org/10.2210/pdb7SQJ/pdb]. The Achromobacter map and model are available as EMD-25382 [https://www.ebi.ac.uk/pdbe/entry/emdb/EMD-25382] and PDB-7SQD [http://doi.org/10.2210/pdb7SQD/pdb].

Field-specific reporting

Please select the one below that is the best fit for your research. If you are not sure, read the appropriate sections before making your selection.

☒ Life sciences ☐ Behavioural & social sciences ☐ Ecological, evolutionary & environmental sciences

For a reference copy of the document with all sections, see nature.com/documents/nr-reporting-summary-flat.pdf

Life sciences study design

All studies must disclose on these points even when the disclosure is negative.

Sample size	Historically 20-100 cells have been used for measuring changes of flagellar waveform as well as other bacterial motility phenotypes (references 5 and 6). For the motility video analysis increasing values of n beyond 30 decreased the p-values even further which told as nothing because the differences we were seeing between our conditions were already highly significant. For analysis of bacterial flagellar filament waveforms we analyzed 37-79 unique flagella waveforms from 25-67 different filaments. This is consistent with previous studies (reference 6).
Data exclusions	No data were excluded.
Replication	Soft agar motility assays were repeated 9 times. Light microscope videos of bacterial motility were repeated at least 3 times from three independent bacterial cultures for each condition.
Randomization	Randomization was not required for structural determination and is not relevant for the other studies which have been done blindly.
Blinding	For soft agar motility assays, no blinding was performed because it is is a simple measurement of distance moved on a bacterial plate. For structural determination blinding is not necessary because given a high resolution structure greater than 4 Å one can accurately model in the correct sequence even if there are similar ones in the genome (see reference 96) or if mass spectrometry identified several potential sequences (this paper). For light microscopy video studies, all movies were mixed together and randomly assigned a number prior to analysis. The analysis of the movies was then done blindly.

Reporting for specific materials, systems and methods

We require information from authors about some types of materials, experimental systems and methods used in many studies. Here, indicate whether each material, system or method listed is relevant to your study. If you are not sure if a list item applies to your research, read the appropriate section before selecting a response.

Materials & experimental systems

n/a	Involved in the study	
☐	☒	Antibodies
☒	☐	Eukaryotic cell lines
☒	☐	Palaeontology and archaeology
☒	☐	Animals and other organisms
☒	☐	Human research participants
☒	☐	Clinical data
☒	☐	Dual use research of concern

Methods

n/a	Involved in the study	
☒	☐	ChIP-seq
☒	☐	Flow cytometry
☒	☐	MRI-based neuroimaging

Antibodies

Antibodies used	The anti-FliC antibody serum was a gift from Howard Berg and Karen Fahrner (this is in acknowledgements) and the secondary is an HRP-conjugated donkey anti-rabbit antibody from Cytiva (NA934)
Validation	The primary has been validated in the lab of Howard Berg.
